# Lignin-Based Epoxy Resins: Unravelling the Relationship between Structure and Material Properties

**DOI:** 10.1021/acs.biomac.0c00057

**Published:** 2020-03-11

**Authors:** Claudio Gioia, Martino Colonna, Ayumu Tagami, Lilian Medina, Olena Sevastyanova, Lars A. Berglund, Martin Lawoko

**Affiliations:** † University of Bologna, Department of Civil, Chemical, Environmental, and Materials Engineering, Via Terracini 28, Bologna, 40131, Italy; ¶ KTH Royal Institute of Technology, Department of Fibre and Polymer Technology, Teknikringen, 56-58, Stockholm, 100 44, Sweden; ∥ Nippon Paper Industries Co., Ltd., Research Laboratory, Oji, Kita-ku, Tokyo, 114-0002, Japan; § Wallenberg Wood Science Center, WWSC, Department of Fiber and Polymer Technology. KTH Royal Institute of Technology, 100 44 Stockholm, Sweden

## Abstract

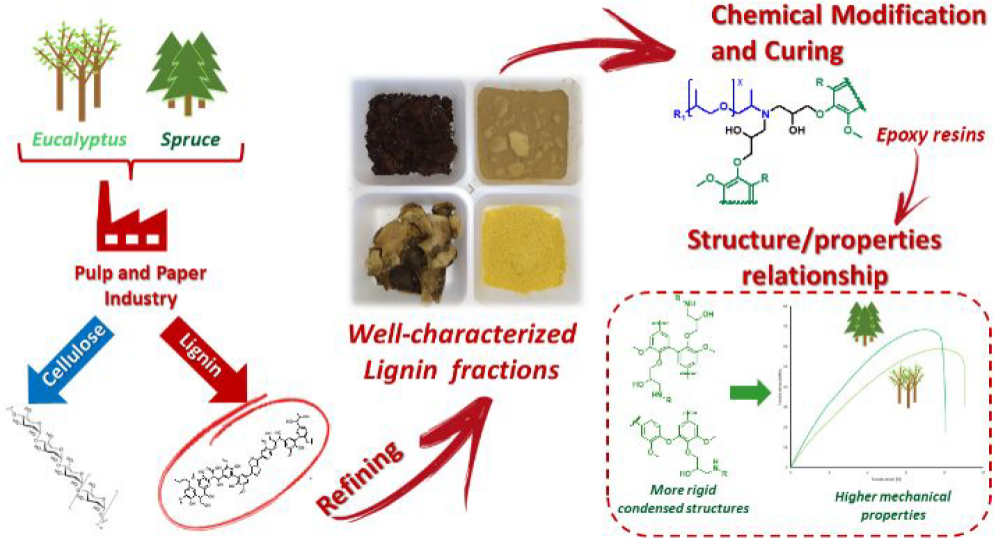

Here we investigate the relationship between thermomechanical properties and chemical structure of well-characterized lignin-based epoxy resins. For this purpose, technical lignins from eucalyptus and spruce, obtained from the Kraft process, were used. The choice of lignins was based on the expected differences in molecular structure. The lignins were then refined by solvent fractionation, and three fractions with comparable molecular weights were selected to reduce effects of molar mass on the properties of the final thermoset resins. Consequently, any differences in thermomechanical properties are expected to correlate with molecular structure differences between the lignins. Oxirane moieties were selectively introduced to the refined fractions, and the resulting lignin epoxides were subsequently cross-linked with two commercially available polyether diamines (Mn = 2000 and 400) to obtain lignin-based epoxy resins. Molecular-scale characterization of the refined lignins and their derivatives were performed by ^31^P NMR, 2D-NMR, and DSC methods to obtain the detailed chemical structure of original and derivatized lignins. The thermosets were studied by DSC, DMA, and tensile tests and demonstrated diverse thermomechanical properties attributed to structural components in lignin and selected amine cross-linker. An epoxy resin with a lignin content of 66% showed a Tg of 79 °C from DMA, Young’s modulus of 1.7 GPa, tensile strength of 66 MPa, and strain to failure of 8%. The effect of molecular lignin structure on thermomechanical properties was analyzed, finding significant differences between the rigid guaiacyl units in spruce lignin compared with sinapyl units in eucalyptus lignin. The methodology points toward rational design of molecularly tailored lignin-based thermosets.

## Introduction

**Figure 1:**
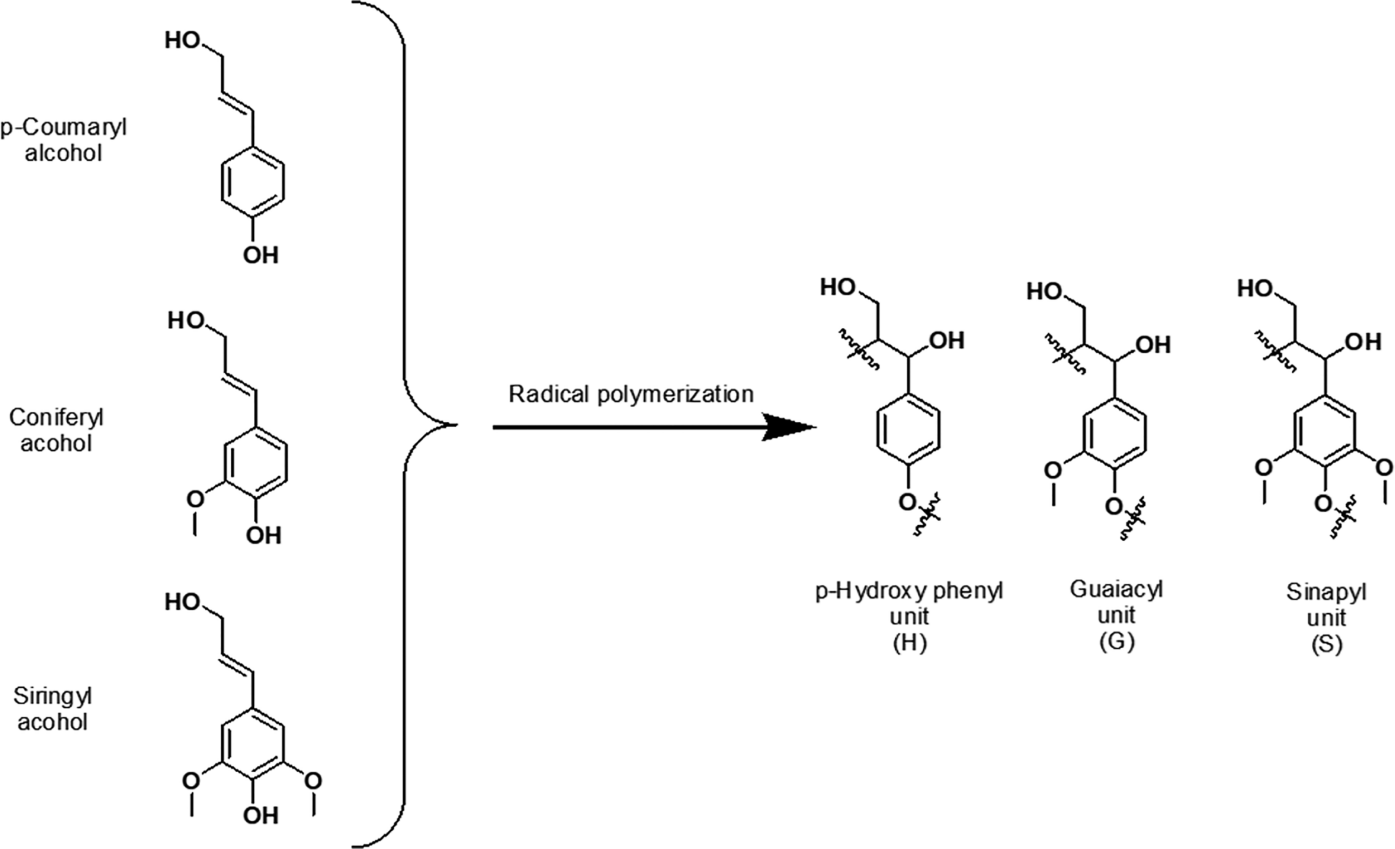
Structures of the main monomeric units composing lignin.

Lignin is one of the three main constituents of wood along with cellulose and hemicellulose. It is the second most abundant biopolymer and most abundant natural aromatic compound.^1,2^ Its biological purpose includes provision of protection and mechanical stability to the plant cell wall and forms a biocomposite with cellulose fibrils and hemicellulose.^3^ Lignin polymerization occurs by way of radical coupling initiated by mild oxidation of the phenolic hydroxyl groups present in the lignin monomers, also called the monolignols. Three main monolignols, namely, *p*-coumaryl, coniferyl, and sinapyl alcohol, constitute the building blocks of lignin and differ in the methoxyl substitution in the ortho position (Figure 1). Depending on the plant species, the monolignol composition of lignin will be different and consequently will the structure of lignin. Softwood lignins are solely formed from coniferyl alcohol, whereas hardwood lignins from both coniferyl and syringyl alcohols, with the former more abundant. Remarkably, the plant cell is able to control the composition of the monomer feed to tune the mechanical properties of the resulting wood tissue.^4^

From an industrial perspective, lignin represents a cheap byproduct of pulp and paper production and currently, it is incinerated to recover the pulping chemicals and energy for the cooking process. Framed in a sustainable development perspective, however, lignin is gaining more and more attention as a valuable feedstock for the substitution of petrol-based derivatives. Strategic fields regarding energy production,^5,6^ building block synthesis,^7,8^ and materials science^9^ efforts to exploit unique lignin structures for new materials. In addition to scientific progress, lignin has been used for nanoparticles^10−12^ and multifunctional materials.^13^ While a first approach toward lignin exploitation aims for its controlled degradation into defined substances suitable to obtain fuels^14,15^ and chemicals,^16−18^ lignin can be used to synthesize novel macromolecular architectures, with favorable effects from the rigid aromatic structures. Although lignin is currently studied for the production of a variety of materials, including hydrogels,^19,20^ composites,^21,22^ and thermoplastics,^23^ one of its most promising applications is related to the fabrication of thermosetting materials such as phenol/formaldehyde resins,^24,25^ polyurethanes,^26,27^ epoxies,^28,29^ and thiol–ene resins.^30^ The presence of functional groups such as carboxylic acids, phenols, and aliphatic alcohols enables the introduction of functionalities suitable for subsequent material syntheses. This aspect, along with its rigid polyaromatic structure, is of interest for novel thermoset polymers. Nevertheless, despite the huge potential of lignin as natural feedstock for polymer and materials systems, a major drawback is the heterogeneity created during both biosynthesis and the extraction processes of lignin.

As previously mentioned, the structure of native lignin is significantly affected by the nature of the monolignols involved in the polymerization. Furthermore, the extraction process applied to retrieve lignin from wood is responsible for major structural modifications favoring the degradation of labile bonds and introducing new chemical bonds. This has both positive and negative implications for material synthesis. The positive is the enhancement in hydroxyl functionality, which meets the prerequisite for the multifunctionality required for the stepwise synthesis of cross-linked networks. The negative is the lack of control in the synthesis of macromolecular structures, which is reflected in poorly reproducible thermomechanical properties with the consequent reduction of the potential of lignin-related materials. Another drawback in most related studies is the lack of thorough structural characterization. Any large-scale material production from lignin will rely on optimized and controlled synthesis, where the control can be quantified. Hence, molecular structure monitoring of all synthesis steps is a prerequisite.

In order to overcome these limitations, efforts have been directed to use more reliable lignin feedstocks in the synthesis of lignin-based thermosetting resins. The organosolv process is a mild extraction method that produces lignin from wood biomass by means of organic solvents extraction. Since the operating conditions usually allow to obtain lignin of relatively low polydispersity, this method has been successfully employed to prepare lignin-based epoxy resins,^31^ although molecular structure details were lacking. Other approaches involve partial depolymerization,^32^ hydrogenolysis,^33^ and hydrolysis^34^ of lignin in order to develop materials based on a more homogeneous feedstock. However, no such lignin is currently available at an industrial scale, and it is produced in a negligible amount compared to industrial processes such as Kraft technology.

In this context, we recently demonstrated the possibility to refine and fully characterize technical lignins by different techniques such as ultrafiltration^35^ and solvent fractionation.^36,37^ Kraft lignin, reportedly the most heterogeneous of technical lignins, yet the most available, was refined to obtain fully characterized fractions with low polydispersity and a molecular weight ranging between 1 to 17.7 kDa. The mechanical behavior of the epoxy resins based on such fractions demonstrated a correlation with the molecular weight of the lignin employed.^38^

With this previous effort, the possibility to tailor the properties of lignin-based materials was introduced. Such development represents only the first step for controlled lignin-based materials and has prompted the necessity for deeper investigations into molecular-scale phenomena contributing to material properties. A detailed understanding of the effect of structure and chemical composition of defined technical lignins from different plant species as well as lignin interaction with different cross-linking agents is still missing. Achieving such information will be critical to the understanding of the performance of lignin-based thermosets on the molecular and macromolecular scale. Such efforts have commenced, in that heterogeneous technical lignin from different biological sources was refined according to a well-established sequential solvent extraction technique.^36^

Such a procedure allows the specific selection of homogeneous lignin fractions of comparable molecular weight and defined by low polydispersity. When such lignins are subjected to chemical modification, the functionalized lignins can be cross-linked to obtain thermosetting resins. Specifically, epoxy resins cured by polyamines represent a desirable target due to their wide range of applications, their easily tunable thermomechanical properties and well-established curing mechanism.^39^

Building on our previous efforts where thermoset properties were correlated with molar mass of the refined lignins,^38^ the objective of this work is to develop a deeper understanding of how parameters such as lignin chemical composition, skeletal structure and functional group distribution, along with the size of the cross-linking agent, influence the material properties. Such knowledge is essential for the development of novel strategies to tailor lignin-based materials toward high-value applications. In addition, we aim to identify lignin-based epoxy compositions of sufficiently high Tg combined with toughness, for consideration in engineering applications such as structural composite materials.

## Experimental Section

### Materials

All chemicals were of analytic grade and used as received unless stated otherwise. Spruce KL and Eucalyptus KL were obtained from the Lignoboost process and washed, before use, with acidic water (pH 2) to remove impurities and dried in a vacuum oven (24 h at 40 °C). Ethyl acetate (EtOAc), ethanol absolute (EtOH), acetone, sodium hydroxide (NaOH, ≥ 98%), epichlorohydrin, HCl, Jeffamine D2000.

### Methods

#### Nuclear Magnetic Resonance

Nuclear magnetic resonance (NMR) (^1^H-, ^31^P-, and 2D heteronuclear single quantum coherence (HSQC)) was recorded at room temperature on a Bruker Avance III HD 400 MHz instrument with a BBFO probe equipped with a Z-gradient coil for structural analysis. Data were processed with MestreNova (Mestrelab Research) using 90° shifted square sine-bell apodization window; baseline and phase correction were applied in both directions. ^31^P NMR samples were prepared and analyzed according to the procedure reported by Argyropolous in 1994.

#### Fourier Transform Infrared Spectroscopy

Transform infrared spectroscopy (FT-IR) was performed using a PerkinElmer Spectrum 2000 FT-IR equipped with a MKII Golden Gate single reflection ATR system of Specec Ltd. All spectra were recorded in the range of 600 to 4000 cm^–1^ with 32 scans averaged at 4.0 cm^–1^ resolution at room temperature. All data were analyzed using PerkinElmer Spectrum software V10.5.1.

#### Differential Scanning Calorimetry

Differential scanning calorimetry (DSC) was conducted using a Mettler-Toledo DSC equipped with a sample robot and a cryo-cooler and evaluated with Mettler Toledo STARe software V15.00a. Unless otherwise stated, the measurements had a heating and cooling rate of 10 K/min and were performed under N_2_ atmosphere.

#### Size Exclusion Chromatography

Size exclusion chromatography (SEC), using a SEC 1260 infinity (Polymer standard service, Germany) equipped with a PSS precolumn, PSS column 100 Å, and PSS GRAM 10 000 Å analytical columns thermostated at 60 °C, was performed to determine the molecular weight and dispersity of the different lignin samples. The detection system included a UV detector in series with a refractive index detector. DMSO + 0.5% LiBr was used as eluent with a constant flow rate of 0.5 mL/min. A calibration plot was constructed with pullulan standards.

#### Tensile Tests and Dynamical Mechanical Thermal Analysis

Tensile tests and dynamical mechanical thermal analysis (DMTA) were performed on cured samples (35.0 × 5.0 × 0.5 mm^3^) conditioned for 100 h at 23 °C and 50% relative humidity (RH) using a Single Column Universal Testing Machine Instron 5944 equipped with a load cell of 500 N and a Q800 DMA apparatus from TA Instruments in three-point bending mode, respectively. The tensile tests were performed according to the ASTM D3013-13 and D638-14. The DMTA measurements were carried out according to the Standard Test Method for Plastics: Dynamic Mechanical Properties: In Flexure (Three-Point Bending) ASTM D5023-07 at a constant frequency (1 Hz), amplitude of 20 mm, a temperature ranges from −100 to 150 °C, and with a heating rate of 3 °C/min. Three replicates were performed for each formulation. The glass transition temperatures (Tg) were determined as the peak of the loss modulus E″ according to the standard ASTM D4092-07 (reapproved 2013).

### Procedures

#### Extraction Procedure

Lignoboost Kraft lignin from Spruce or Eucalyptus were extracted by organic solvents to obtain soluble lignin fractions.^36^ Eucalyptus Kraft lignin (10 g) and EtOAc (100 mL) were introduced into a round-bottom flask equipped with magnetic stirring. The suspension was stirred at room temperature. After 2 h, the insoluble particles were removed from the mixture by filtration and the remaining solution was dried under reduced pressure producing EF_1_. The filtrated insoluble lignin was furthermore extracted with EtOH producing EF_2_. The residual material was finally separated by filtration. All the obtained extracted fractions were retrieved by a freeze-drying procedure to obtain brown powders. Spruce Kraft lignin was extracted with EtOAc following the same protocol to obtain fraction SF.

#### Lignin Modification

In a round-bottom flask equipped with magnetic stirrer were introduced the respective lignin fraction (500 mg), a mixture of water and acetone (50%v/v, 75 mL), NaOH (3 eq. the number of active OH of the lignin fraction), and epichlorohydrin (20 eq. the number of active OH of the lignin fraction). The mixture was stirred at 50 °C for 5 h. Afterward, the reaction was quenched by introducing 50 mL of water and lowering the pH to 3.5 with HCl 0.1 M. The resulting precipitated product was recovered by filtration on a glass filter (pore size 4). Such filtrate was washed with 2 portions of deionized water to remove traces of acid. Finally, the product was dissolved in acetone and precipitated with deionized water to obtain a homogeneous water dispersion. The mixture was finally freeze-dried to obtain a brown powder.

#### Synthesis of the Thermosetting Resins

In a vial were introduced 30 mg of epoxidated lignin fraction and an acetonitrile solution of the corresponding amount of Jeffamine D2000 to obtain a homogeneous solution. The mixture was then cast in a Teflon mold and treated at 50 °C for 1 h to remove the solvent. Afterward, the mixture was cured successively 2 h at 100 °C and 2 h at 150 °C.

## Results and Discussion

### Fractionation and Characterization of Technical Lignins

Kraft lignin derived from two different sources, eucalyptus and spruce, were used. The rationale for this choice was that these two species are expected to produce structurally different Kraft lignins based on their monolignol compositions.

Fractionations by molar mass were carried out by means of sequential solvent extraction with benign and safe solvents so that reproducible, low polydispersity fractions were obtained of fairly low molar mass.^36^ Following the fractionation, we sought to identify fractions from the refined spruce and eucalyptus Kraft lignins that had comparable molecular weights. Three fractions met this criterion (Table 1); EF_1_ and EF_2_ refer to eucalyptus Kraft lignin fractions obtained by extraction, respectively, with ethyl acetate (35% of yield) and ethanol (33% of yield), while SF is a spruce Kraft lignin ethyl acetate fraction (25% of yield). These two wood species show significant differences in the native lignin structure. Such differences will carry over to the technical lignins.

**Table 1:** Molecular Weight and Functional Groups of the Lignin Fractions

	EF_1_	EF_2_	SF
Mn (g/mol)^a^	700	900	700
Mw (g/mol)^a^	1050	1440	1120
PDI^a^	1.5	1.6	1.6
aliphatic OH (mmol/g)^b^	0.7	1.6	0.9
carboxylic acid (mmol/g)^b^	0.3	0.4	0.7
noncondensed phenols (mmol/g)^b^	1.2	1.1	3.1
C5-substituted phenols^c^ (mmol/g)^b^	3.9	3.4	1.8
total phenols (mmol/g)^b^	5.1	4.5	4.9

a Estimated by SEC analysis.

b Calculated by ^31^P NMR.

c For EF1 and EF_2_ the substitution at C5 mainly constitutes a methoxy group yielding sinapyl phenols (see Figure 1). For SF, the substitution constitutes a lignin moiety giving rise to so-called C5 condensed structures (e.g., phenolic 5–5′, 4-O-5′)

SEC analysis evinced that EF_1_ and SF show the same molecular weight (700 g/mol), while EF_2_ reported a slightly higher Mn (900 g/mol). In all the selected samples, a good homogeneity with respect to molar mass was achieved, as confirmed by the low PDI values demonstrated (Table 1). ^31^P NMR was applied for qualitative and quantitative analysis of reactive hydroxyl functionalities in the lignins (Figures S2–S4). EF_1_ and SF present the highest amount of total phenolic hydroxyl contents, respectively, 5.1 and 4.9 mmol/g (Table 1). Importantly, EF_1_ and EF_2_ have a higher content of sinapyl-based phenolics while SF phenolics are almost entirely guaiacyl-based (see Figure 1). These differences originate from the monolignol composition in native lignins; hardwood species are dominated by sinapyl units while softwood lignin consists entirely of guaiacyl units. Sinapyl-based lignin does not form lignin interunit coupling at C5 because this position is occupied by a methoxy group. Therefore, the extent of C5 condensed phenolics in eucalyptus is significantly lower than in spruce. Aliphatic alcohols are mainly preserved in EF_2_ while SF shows the highest amount of carboxylic acids, proving to be more subjected to oxidative processes during Kraft pulping.

**Figure 2:**
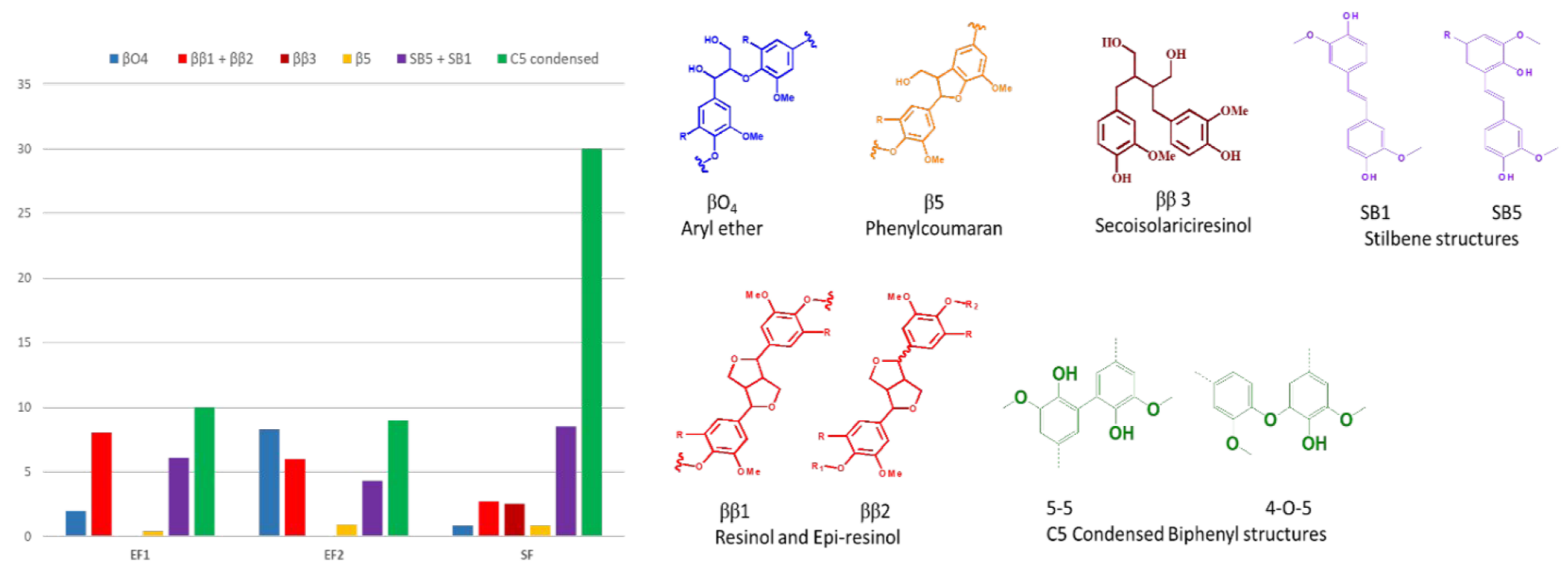
Semiquantification of lignin interunits presented for the pristine eucalyptus Kraft lignin, the refined fractions obtained from it, and the low molar mass spruce Kraft lignin fraction.

Further details on lignin skeletal structure were obtained by two-dimensional NMR, specifically ^13^C–^1^H HSQC, which, unlike 1D-NMR techniques for lignin analysis, resolves overlapping signals in both proton and carbon dimensions, consequently unveiling detailed molecular structure. Typical HSQC spectra of the Kraft lignin fractions are shown in Supporting Information (Figure S6–S8), and the data obtained from them are in Figure 2. The aryl ether (βO4), phenylcoumaran (β-5), and resinol structures (ββ_1_ and ββ_2_) are native lignin structural elements, but the amount of these structures is severely reduced in technical lignins due to chemical reactions of lignin during Kraft pulping.^40−42^ For instance, through elimination reactions, the βO4 structure yields enol ether structures (Figure 2), while ether bond cleavage generates new phenolic ends as observed by the ^31^P NMR analyses (Table 1). The ββ and β-5 linkages are stable but react with the elimination of formaldehyde to form stilbene structures. In fact, part of the resinol structure remains intact and simply epimerizes at the reaction conditions.^42^

In addition, ^13^C-APT NMR analysis was applied particularly to EF_1_ with the specific aim of analyzing the C5 condensed structures, whose detection by ^31^P NMR was compromised by overlap with sinapyl phenolics. These signals appear as quaternary carbons at 132.5 ppm in the spectra (Figure S9).

Based on such detailed analyses by 2D-NMR and ^31^P NMR (Table 1, Figure 2, Table 2), a qualitative molecular level description of the fractions can be obtained. The major differences between the SF and EF samples are the dominance of C5-condensed structures (see Figure S2–S4 for ^31^P NMR spectra) which was about 3-fold higher than the amount in the former. Furthermore, the SF fraction has about twice as many stilbene structures than the EF_1_. Interestingly, both condensed C5 and stilbene structures provide high aromatic density and low molecular mobility. Stiffness results from the lack of free rotation between constitutional monomers and the aromatic density results from direct couplings of aromatic rings through C–C bonding at position C5. The monolignol composition of lignin may significantly affect its properties. A hint of the relative methoxyl content in the fractions can be obtained from the syringyl to guaiacyl ratio (S/G ratio, Table 2), and is consistent with the preservation and dominance of S-units in eucalyptus Kraft lignin fractions, while guaiacyl units are the main constituents of spruce Kraft lignins.

**Table 2:** Semiquantification of the Detected Connecting Units for the Lignin Fractions

connecting units (%)^a^	EF_1_	EF_2_	SF
βO4	2	8.3	0.8
ββ_1_ + ββ_2_	8	6	2.7
ββ_3_	--	--	2.5
β5	0.4	0.9	0.8
SB_5_	2.9	3.1	5.7
SB_1_	3.2	1.2	2.8
C5 condensed	10	9	30
S/G ratio^b^	3.3	2.5	no S-units
Tg (°C)^c^	82	147	75

a Estimated by HSQC analysis.

b Syringyl to guaiacyl ratio.

c Calculated by DSC analysis.

### Chemical Modification and Curing Reaction

Epoxy functionalities were selectively introduced on the fractions by reacting with epichlorohydrin in acetone and water mixture under mild conditions. Specifically, EF_1_ and EF_2_ were modified into the corresponding eucalyptus epoxies EE_1_ and EE_2_, while SF was transformed into spruce epoxy-lignin SE. The ^31^P NMR analysis confirmed that, at the optimized condition of reaction, only phenols and carboxylic acid sites undergo chemical modification, leaving aliphatic alcohols unreacted (see SI, Figure S15 for ^31^P NMR spectrum). HSQC analysis of EE_1,2_ and SE identified peaks assigned to the added functional oxirane group, respectively, at 70/4.4, 70/3.8, 50/3.3, and 45/2.4 ppm. Furthermore, no major structural modification other than epoxidation was observed, underlining the mild condition of reaction, and the maintenance of the structural integrity of the lignin skeleton. The extent of modification was assessed by ^1^H NMR in the presence of 4-nitrobenzaldehyde as internal standard in order to determine a stoichiometric amount of cross-linker for the curing step (Figures S16–18). As reported in Table 3, all the modified lignin fractions present a comparable epoxy content, comprised between 4.8 and 5.1 mmol of epoxy functionalities per gram of lignin. These values are comparable to industrially available bisphenol A based epoxy prepolymers (DGEBA) with epoxy content in the range of 5.3 mmol/g,^43^ and such modified lignins represent a potential renewable alternative.

**Table 3:** Formulation of the Synthesized Thermosets

resin	epoxidated fraction	epoxy groups (mmol/g)^a^	*n̅*^b^	cross-linker	lignin content (w/w%)	Tg (°C)^c^	ΔCp (J/g°C)^c^	Tα^d^
ET_1–2000_	EE_1_	5.1	3.6	JD2000	30	–49	0.42	–47
ET_2–2000_	EE_2_	5.1	4.6	“	31	–51	0.28	–53
ST_2000_	SE	4.8	3.3	“	33	–50	0.37	–52
ET_1–400_	EE_1_	5.1	3.6	JD400	64	73	0.37	35
ET_2–400_	EE_2_	5.1	4.6	“	66	n.d.	n.d.	89
ST_400_	SE	4.8	3.3	“	66	60	0.58	79
Araldite GY 6010^e^	DGEBA	5.3	2.0	“	0 (66 w/w% of DGEBA)	53	n.r.	n.r.

a Determined by ^1^H NMR;

b Average number of epoxy groups available for each macromolecule;

c Determined by DSC;

d Determined by DMA;

e All the data are obtained from the technical data sheet.^43^

Additionally, the average number of epoxy groups available for each macromolecule (*n̅*) can be estimated according to the following equation:
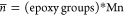


In this case, the modified fractions from eucalyptus, EE_1_ and EE_2_, demonstrate a higher number of reacting sites in comparison with the modified spruce lignin while DGEBA can only present 2 epoxy groups per molecule.

**Figure 3:**
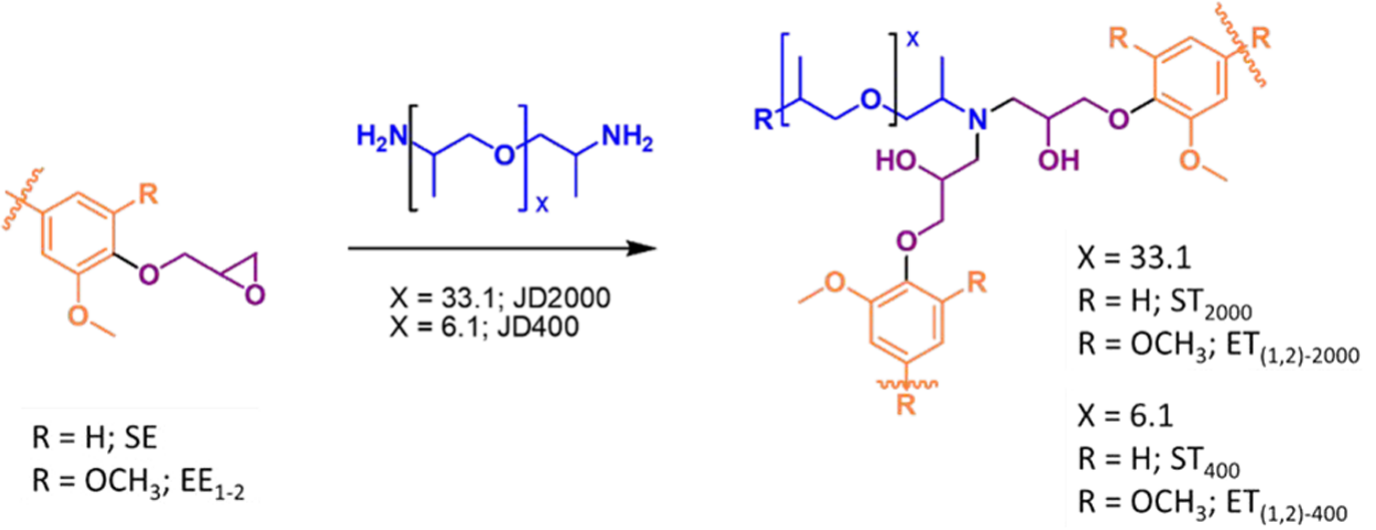
Chemical modification of lignin fractions and curing to obtain thermosetting resins.

As reported in Table 3, a pool of six different thermosets was prepared from the epoxidated lignin fractions (Figure 3) and designed to investigate how different structural features contribute to thermomechanical properties of the materials.

Two poly(propylene oxide) diamines, namely Jeffamine D2000 and Jeffamine D400, were selected as cross-linking agents for the epoxidated lignin fractions. Such compounds are industrially available and potentially deriving from biobased propylene oxide.^44^ The amount of cross-linking agent was selected at the stoichiometric ratio with the number of epoxy functionalities on the lignin prepolymers. All the thermosets were obtained by solvent casting and cured by thermal treatment.

The effect of the structure of the lignin segment was initially estimated by comparing the properties of ET_1–2000_, ET_2–2000_, and ST_2000_, obtained respectably from EE_1_, EE_2_, and SE, cured with Jeffamine D2000. An aliphatic amine of such high Mn was specifically chosen to confer ductility to the material by balancing the inherent rigid aromatic structure of lignin. A shorter cross-linking agent, instead, is expected to reduce ductility and possibly to increase the Tg of the thermoset by enhancing the contribution of the aromatic structure of lignin. Jeffamine D400 was selected to cure EE_1_, EE_2_, and SE to obtain ET_1–400_, ET_2–400_, and ST_400_, respectively. Since the cross-linkers present a different ratio of amine groups/molecular weight, the relative content of the lignin prepolymer and amine curing agent was strongly influenced. Accordingly, the content of lignin does not exceed 33% for the JD2000 related materials, while it is twice as high (66%) for the thermosets cured with JD400 (Table 3). Such lignin-based materials can be compared with a family of commercially available, fully petrol-based thermosets such as Araldite GY 6010 (Table 3),^43^ presenting a similar amount of lignin-based epoxy prepolymer (66%) and cured with Jeffamine D400.

### Thermoset Structure–Property Relationships

The thermal behavior of the thermosets was studied by DSC analysis. All the samples cross-linked with Jeffamine D2000 (ET_(1,2)-2000_ and ST_2000_) present only one distinctive Tg, in the range between −49 °C and −52 °C (Figure S20). In this case, the Tg is mainly governed by the long polyether chain of the aliphatic amine cross-linker. As a consequence, the nature of lignin does not impart any significant thermal effect as demonstrated by comparing ST_2000_ with ET_1–2000_. Most likely, these thermosets are two-phase materials with lignin- and polyether-rich domains. By decreasing the length of the diamine, the contribution of lignin is predominant and the Tg increases. ET_1–400_ and ST_400_ show Tg of 73 and 60 °C, respectively (Figure S21). Such range of thermal properties, in fact, demonstrate that these materials overtake the thermal behavior of the previously mentioned commercially available Araldite GY6010, with a Tg of 53 °C,^43,45^ opening for attractive renewable alternatives based on lignin. One may note that the tensile strengths of ET_1–400_, ET_2–400_, and ST_400_ are higher than for DGEBA cured with the same curing agent.^46^ Strain to failure is lower for the present lignin-based epoxies, which is expected due to the more heterogeneous structure of the lignin epoxide and the final thermoset network.

**Figure 4:**
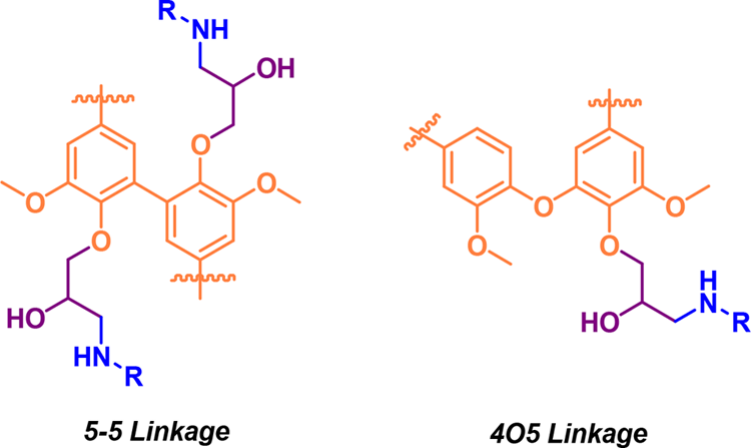
Examples of condensed connecting units typical for spruce Kraft lignin.

**Figure 5:**
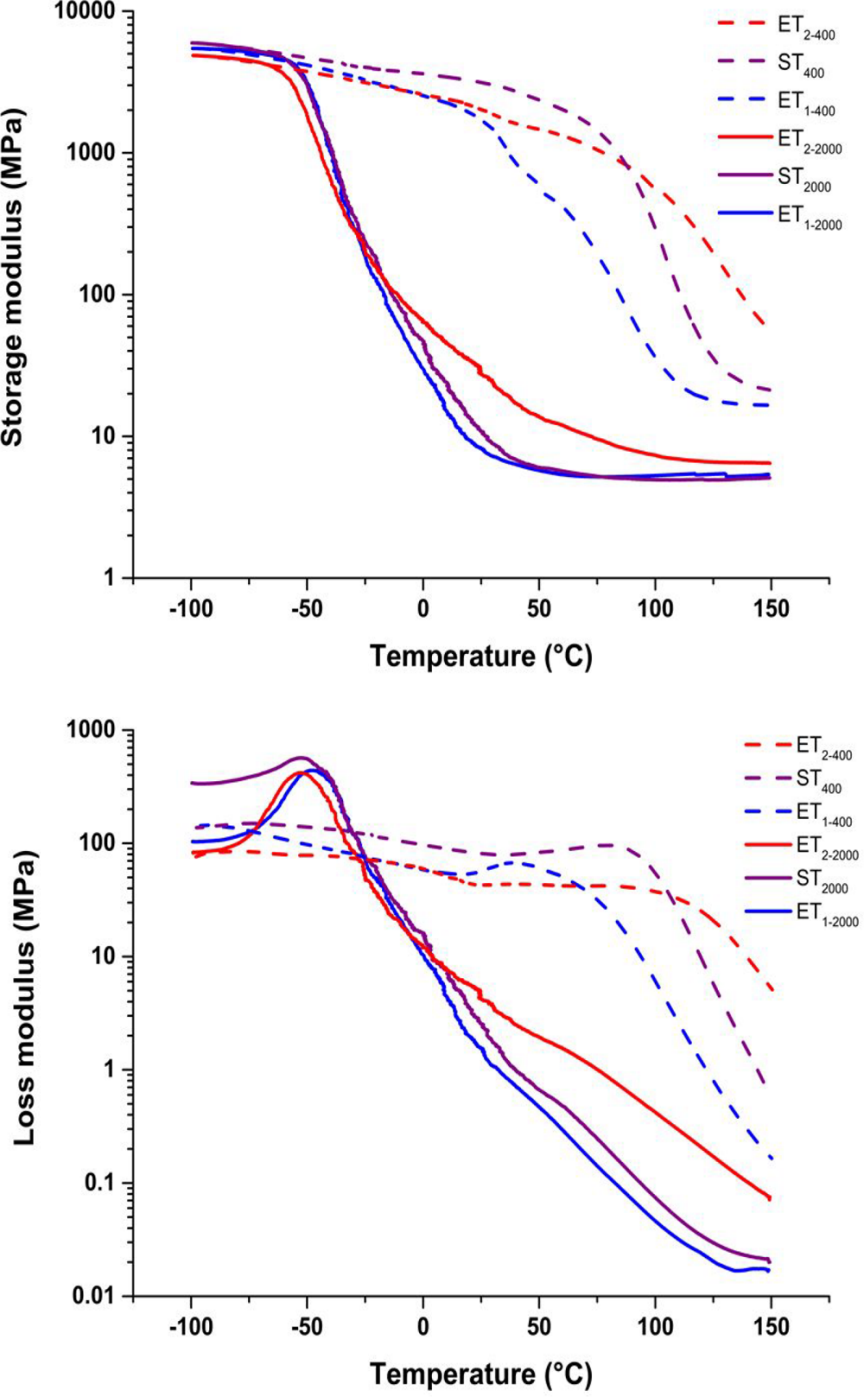
DMA analysis of the thermosets. Storage modulus (top), loss modulus (bottom) as a function of temperature at a frequency of 1 Hz. Note that epoxy resin designations are clarified in Table 3.

The Tg transition of ET_2–400_ is broad and difficult to evaluate by DSC, although the storage modulus versus temperature curve in Figure 5 indicates that Tg is slightly higher than for ET_1–400_. The broad transition means that the thermoset network is heterogeneous in nature. Further thermomechanical properties were evaluated by DMA analysis. Tα values (Table 3), obtained as the peak of the loss modulus, were reported for samples ET_(1,2)-2000_ and ST_2000_. These results are in line with the values of Tg obtained by DSC, with values comprised between −47 and −53 °C. Such tendency confirms that long polyether cross-links dominate the thermomechanical properties. Materials such as ET_1–400_, ET_2–400_, and ST_400_, instead, present broad α transitions with values of 35, 89, and 79 °C, respectively. It means the miscibility of lignin and the amine component is such that we possibly have a molecularly mixed one-phase system. In this case, lignin dominates thermomechanical properties. Interestingly, despite a similar molar mass as ET_1–400_, the ST_400_ thermoset presents a much higher Tα value than ET_1–400_ and comparable with ET_2–400_. This effect is most likely due to the unique structural features of ST lignin, with a rigid structure of high aromatic content with hindered, condensed C5 linkages able to prevent free rotation of the macromolecular structure (Figure 4). Such C5 condensed structures (5–5′ interunits) are present in native softwood lignins and stable during pulping. However, new types (1–5′ interunits), recently identified by Lawoko and co-workers^47^ are also formed through radical condensation reactions during Kraft pulping. In contrast, hardwood lignins are dominated by S-units, and the presence of methoxy group at C5 position confers flexibility rather than rigidity.

Cross-link density represents a key parameter to describe and justify the thermomechanical behavior of cross-linked materials. Despite the presence in literature of several methodologies,^48−50^ none of them was suitable for an estimate of the cross-link due to the inherent variability of resin components and the deviation of mechanical properties from an ideal rubber behavior. The common assumption of such theories, in fact, is to consider the chemical structure of the thermosets homogeneous and comparable. However, the epoxidized lignin fractions vary in chemical composition and functionality. For this reason, the parameter *n̅* (Table 3) was introduced to estimate the effect of epoxy functionalization on the material properties.

An overview of the thermomechanical behavior of the resins can be obtained by analyzing DMA data (Figure 5) and tensile tests (Table 4). Figure 5 shows high thermomechanical stability for ET_2–400_, which is characterized by a high value for *n̅*. This should correspond to higher cross-link density.

**Table 4:** Mechanical and Thermal Analysis of the Thermosetting Materials

	DMA analysis			tensile test		
resin	E′ (−100 °C) [MPa]	E′ (20 °C) [MPa]	E′ (100 °C) [MPa]	E_young_ [MPa]	σ_UTS_ [MPa]	ε_break_ [%]
ET_1–2000_	5400	9	5	3.4 ± 0.2	1.0 ± 0.1	42 ± 4
ET_2–2000_	4900	34	7	5.7 ± 0.2	1.4 ± 0.1	39 ± 2
ST_2000_	5900	13	5	6.0 ± 0.1	1.2 ± 0.1	47 ± 2
ET_1–400_	5400	1900	35	1.4 × 10^3^ ± 0.1	59 ± 2	9.8 ± 0.4
ET_2–400_	4900	2200	570	1.6 × 10^3^ ± 0.1	56 ± 1	10.5 ± 3.0
ST_400_	5900	3200	282	1.7 × 10^3^ ± 0.1	66 ± 2	7.9 ± 0.2
Araldite GY 6010^43^	n.r.	n.r.	n.r.	2.9 × 10^3^	58	3.8

a All the data are obtained from the technical data sheet.^43^

The results in Table 4 show good correlation between the modulus measured by tensile tests (Young’s modulus) and measured by DMA (storage modulus) at 20 °C for resins based on lignin from the same type of wood species (eucalyptus hardwood or spruce softwood).

The lower the molecular weight of the corresponding lignin fraction, the higher the modulus in the glassy state of the cured network, in agreement with the results reported in our previous paper.^38^ This may be related to lower free volume in thermosets from lower molar mass lignin.

By comparing resins cross-linked with Jeffamine 2000 and 400, a significantly higher Young’s modulus is noted at room temperature for resins from Jeffamine 400, with shorter cross-linker and higher epoxidized lignin content. This is because of the higher Tg of the Jeffamine 400 resins with higher lignin content (twice as high as for resins made with Jeffamine 2000). The comparison of resins based on lignin from different types of wood shows that spruce-based lignins result in somewhat higher modulus compared to eucalyptus-based resins. Spruce-based lignin has more condensed structures, with reduced molecular mobility compared with eucalyptus analogues. Additionally, ET_1–400_, ET_2–400_, and ST_400_ thermosets can be compared with Araldite GY6010, with a similar epoxide prepolymer content. The petroleum-based Araldite GY6010 has a Young’s modulus of 2.9 × 10^3^ MPa, σ_UTS_ of 58 MPa and ε_break_ of 3.8%.^43^ The lignin-based resins show a lower Young’s modulus but comparable strength σ_UTS_ and a higher ε_break_, with more pronounced ductile behavior (Figure S23). For resins cured with Jeffamine 2000, increased molar mass of the epoxidized lignin increases the ultimate strength σ_UTS_ at room temperature because of the Young’s modulus increase at 23 °C. No significant differences are observed for strain to failure for resins cured with Jeffamine 2000 (Figure S22), and Tg is similar for all three resins, see Table 3.

## Conclusions

Despite their inherent chemical and macromolecular complexity, technical lignin from eucalyptus and spruce were refined into well-characterized fractions and used for the preparation of lignin-based epoxy resins. Mild epoxidation of lignin was carried out followed by curing with flexible, commercially available polyetheramines. By relating information from structural analysis of lignin to thermomechanical properties of the corresponding thermosets, the relationships between the molecular lignin structure and thermoset properties were evaluated.

The lignin structure is of major importance for resins with 66% lignin, which are of great interest as ecofriendly high-performance epoxies based on renewable resources. By comparing fractions with similar molecular weight and reactive centers (epoxy equivalent weight), the contribution of the lignin connecting units to the thermomechanical properties on the molecular level is deduced. Softwood spruce Kraft lignin provides somewhat better thermomechanical properties compared with eucalyptus-based resins. The presence of unique guaiacyl units leads to the formation of more C5-condensed aromatic units, which reduces molecular mobility of this unit. In contrast, hardwood Kraft lignin is dominated by sinapyl units with lower content of condensed units but higher content of flexible methoxy units.

The thermomechanical behavior was shown to reach the property range of commercially available petroleum-based epoxy resins. With a short diamine, such as JD400, the thermomechanical properties were largely lignin-controlled, since the aromatic nature of the lignin increased Tg to a commercially feasible range. In addition, the tensile strength reached 66 MPa and was slightly higher for the spruce-based lignin epoxy resin. Since brittleness is a problem with heterogeneous high Tg thermoset networks from lignin, it is interesting that this thermoset showed unusually high toughness with a strain to failure of 8%; which is about twice as much as for the commercial reference material.

The results show correlations between the molecular structure of technically available lignins and the properties of lignin-based epoxy resins. Such knowledge is essential for the rational exploitation of lignin as a component in new thermosets based on renewable resources. The lignin-based epoxy-amine system is attractive not only in terms of excellent property potential but also in that the curing chemistry shows few side-reactions so that the thermoset formation mechanisms can be analyzed.

## Supporting Information Available

SEC analysis of the obtained fractions, ^31^P NMR analysis of the obtained fractions, HSQC analysis of the obtained fractions, representative ^13^C-APT NMR analysis, DSC analysis of the obtained fractions, HSQC of the epoxidated fractions, ^31^P NMR analysis of the epoxidated fractions, ^1^H NMR analysis of the epoxidated fractions, representative FTIR analysis, DSC analysis of the thermosets, tensile tests (PDF)
