# Supplementary material for: Lignin-Based Epoxy Resins: Unravelling the Relationship between Structure and Material Properties
Source: Biomacromolecules. 2020 Mar 11;21(5):1920–8. doi: 10.1021/acs.biomac.0c00057 (PMC7997103; doi:10.1021/acs.biomac.0c00057)
Supplement: Supplementary file 1 — bm0c00057_si_001.pdf [file bm0c00057_si_001.pdf]

Supporting information

Lignin fractionation.

SEC Analysis

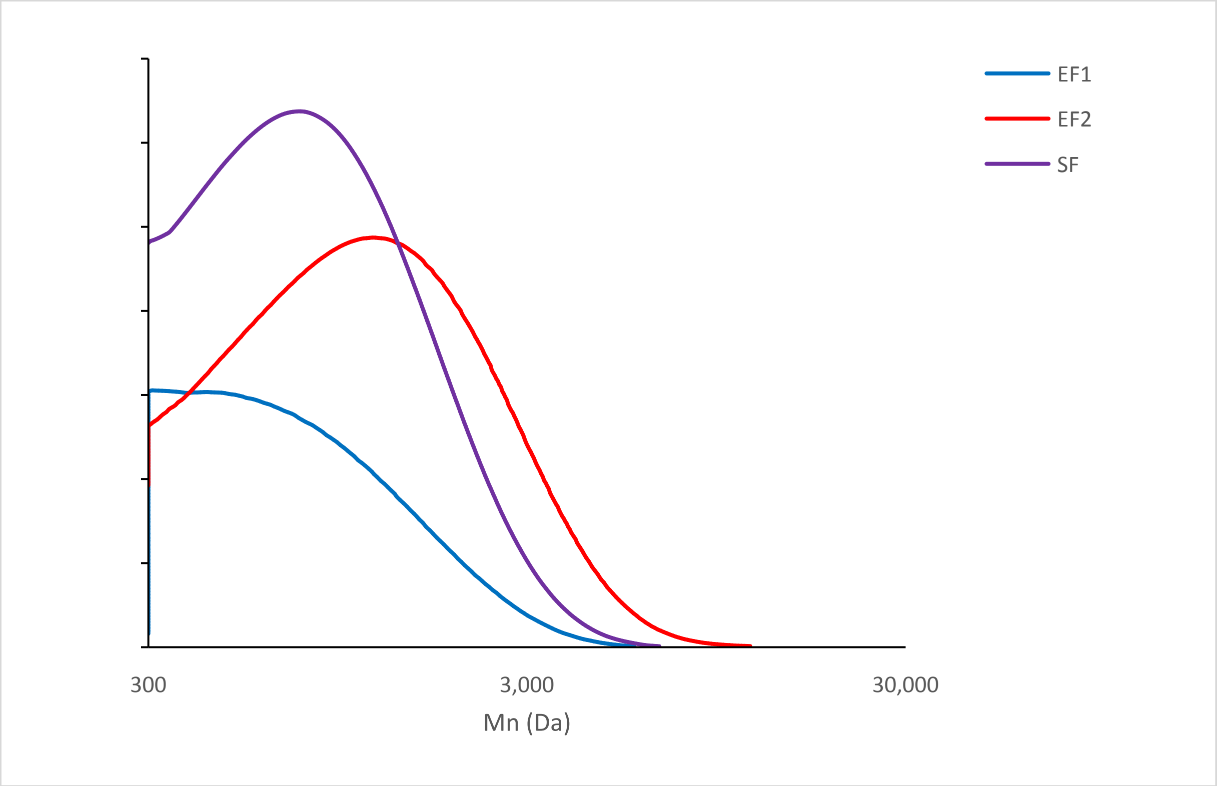

Figure S1: SEC analysis of the obtained lignin fractions.

^{31}P-NMR ($CDCl_3$)

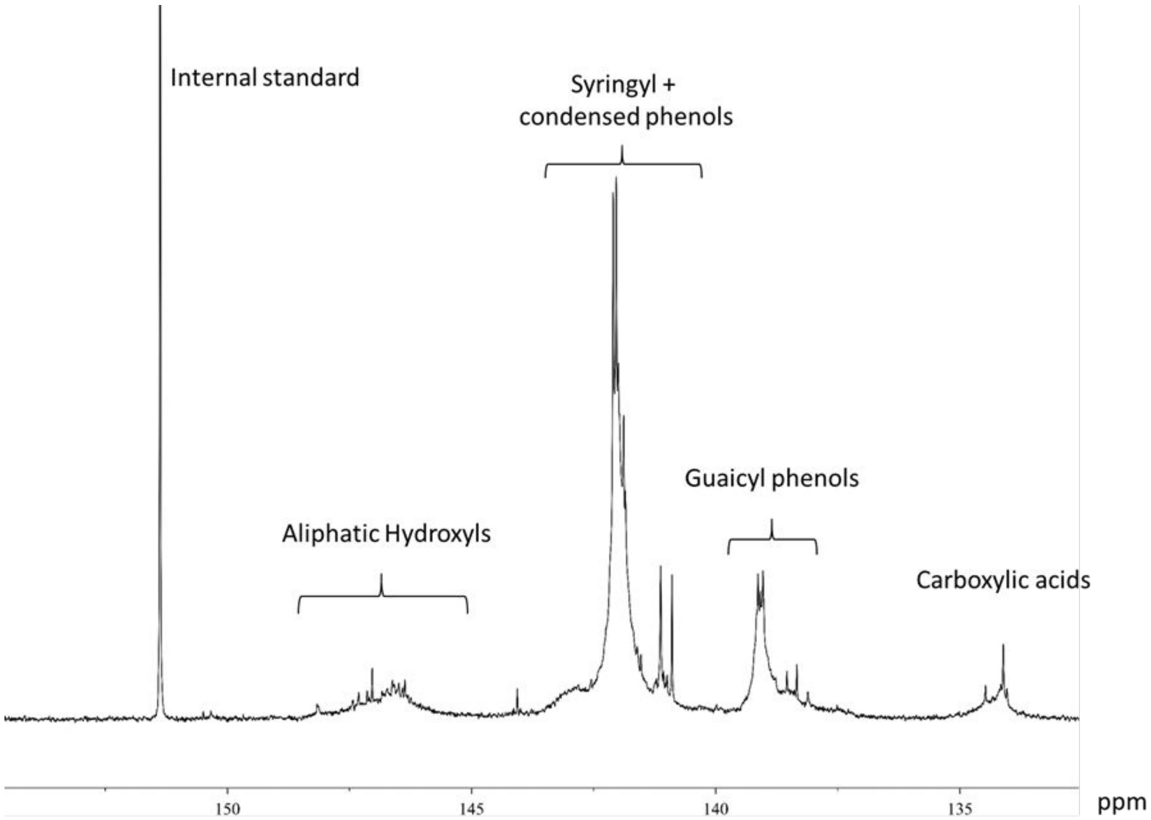

Figure S2: ^{31}P-NMR of the eucalyptus EtOAc fraction (EF_1).

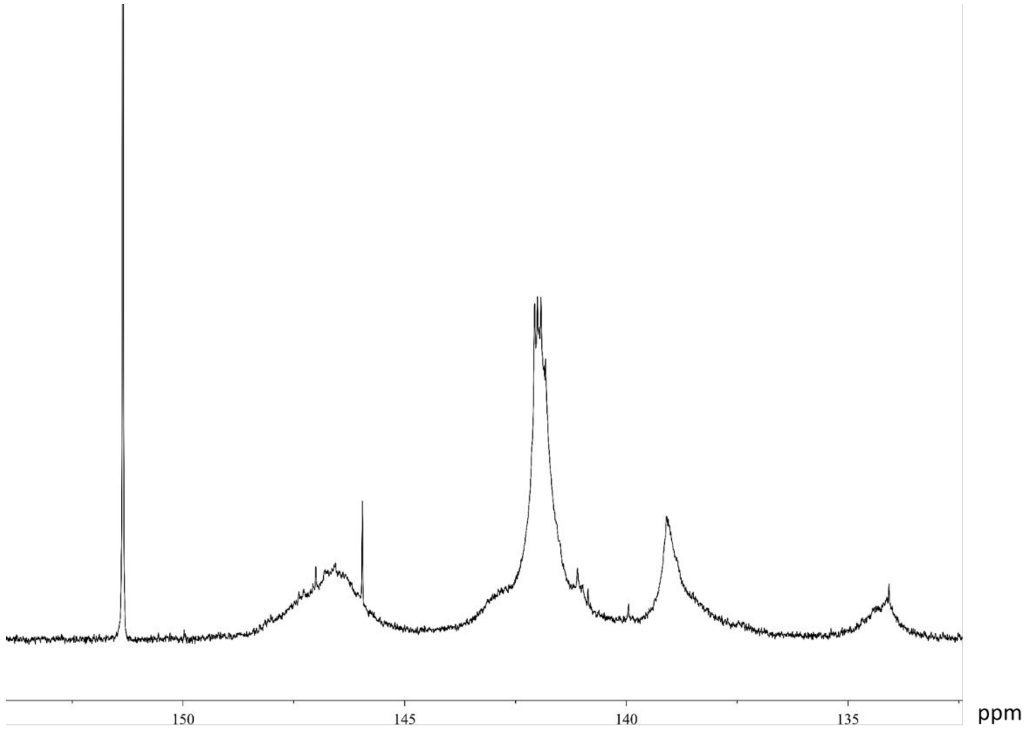

Figure S3: ^{31}P-NMR of the eucalyptus EtOH fraction (EF_2).

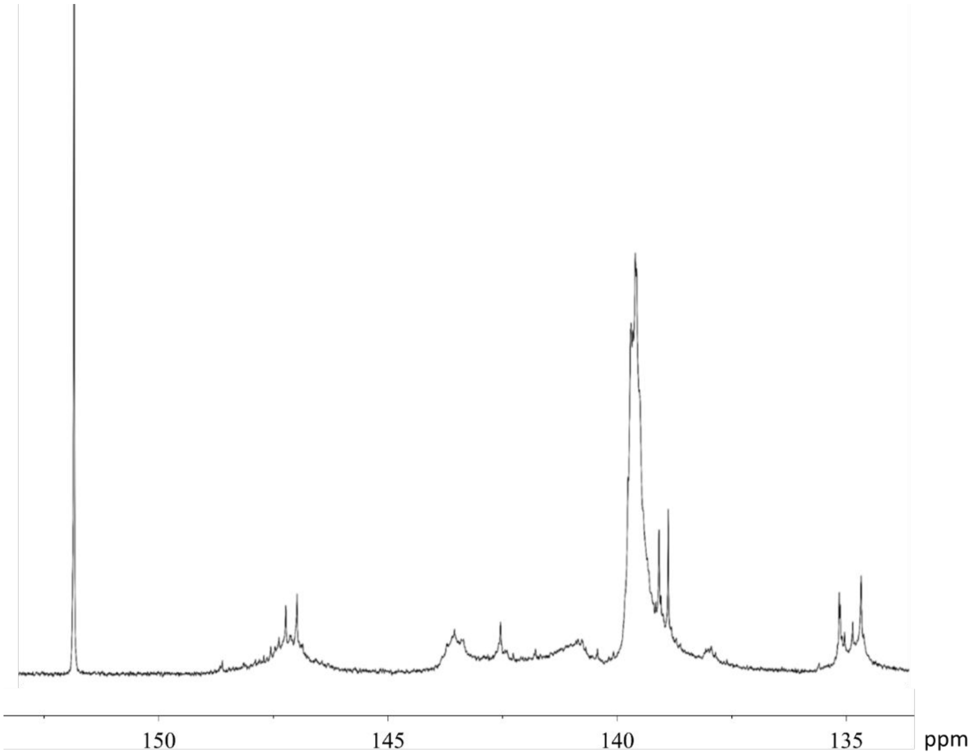

Figure S4: ^{31}P-NMR of the spruce EtOAc fraction (SF).

HSQC Analysis (DMSO)

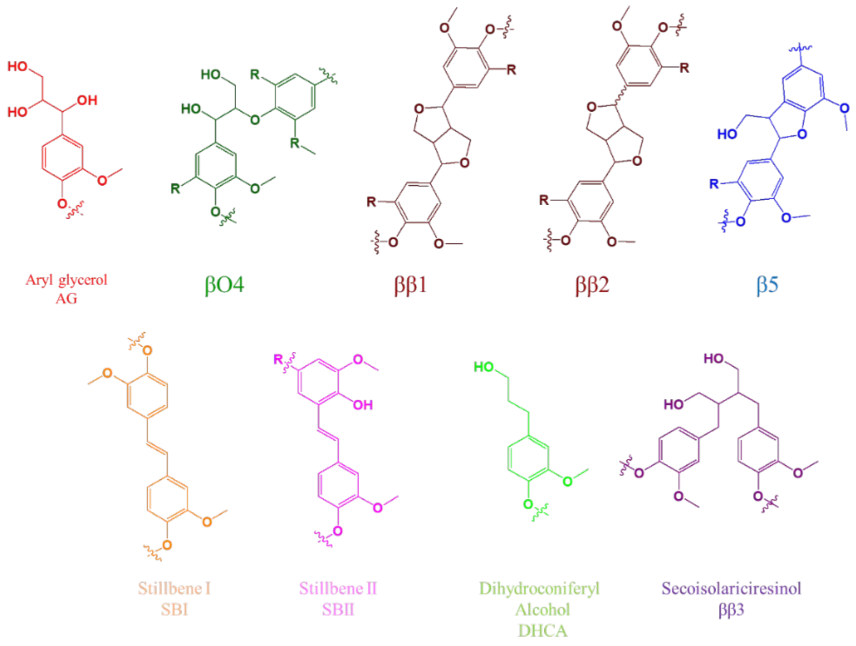

Figure S5: Inter-unit linkages observed in the fractions.

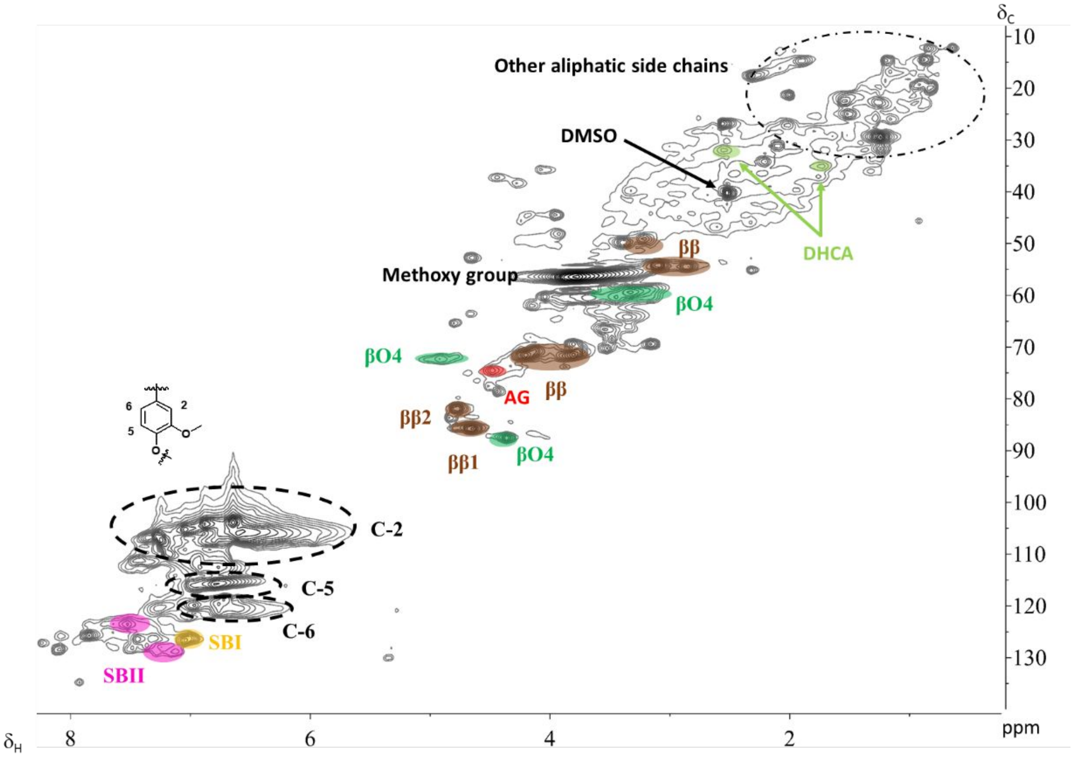

Figure S6: HSQC analysis of the eucalyptus EtOAc fraction (EF_1).

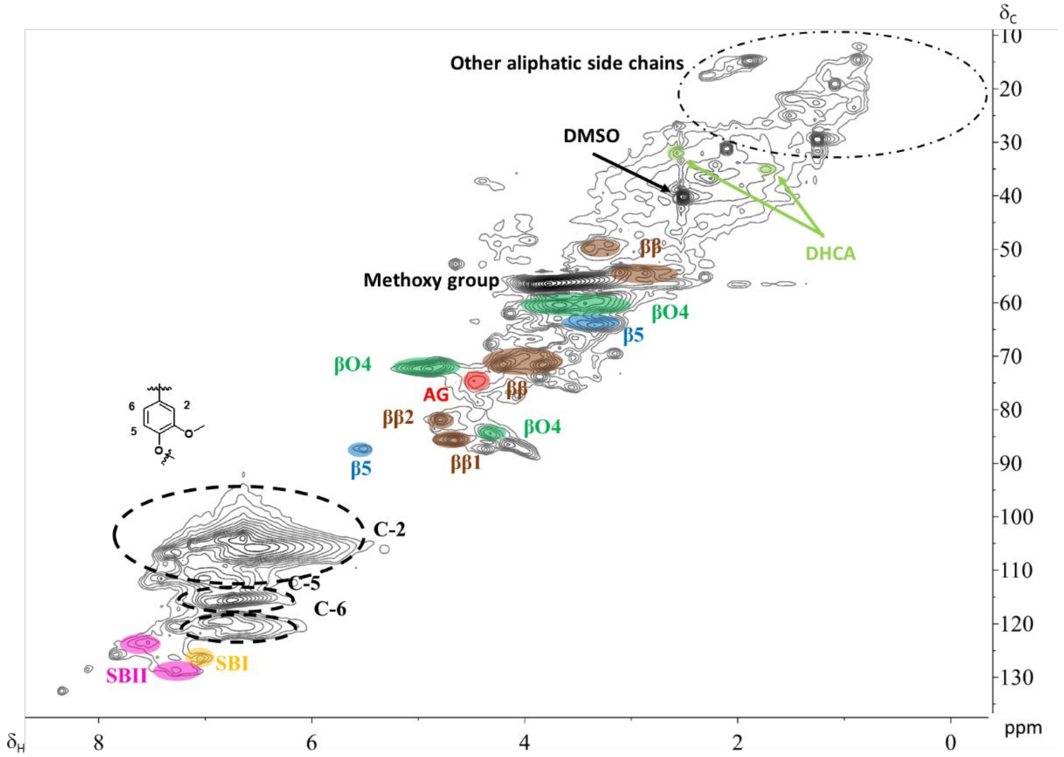

Figure S7: HSQC analysis of the eucalyptus EtOH fraction (EF_2).

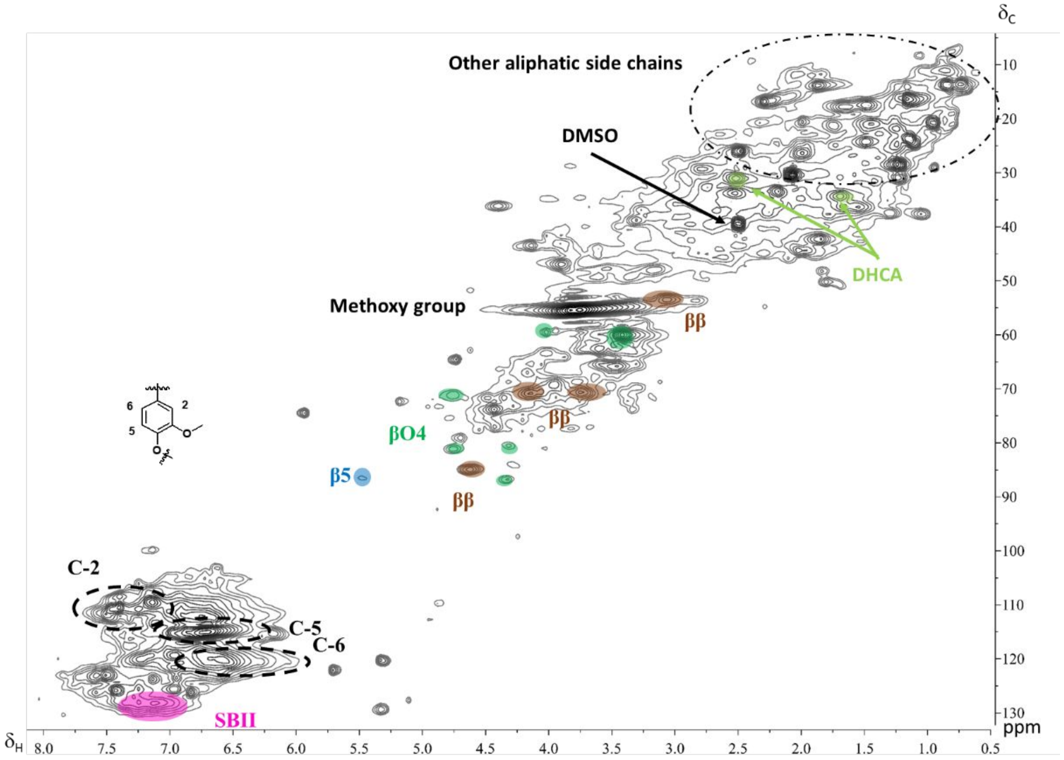

Figure S8: HSQC analysis of the spruce EtOAc fraction (SF).

^{13}C-APT NMR

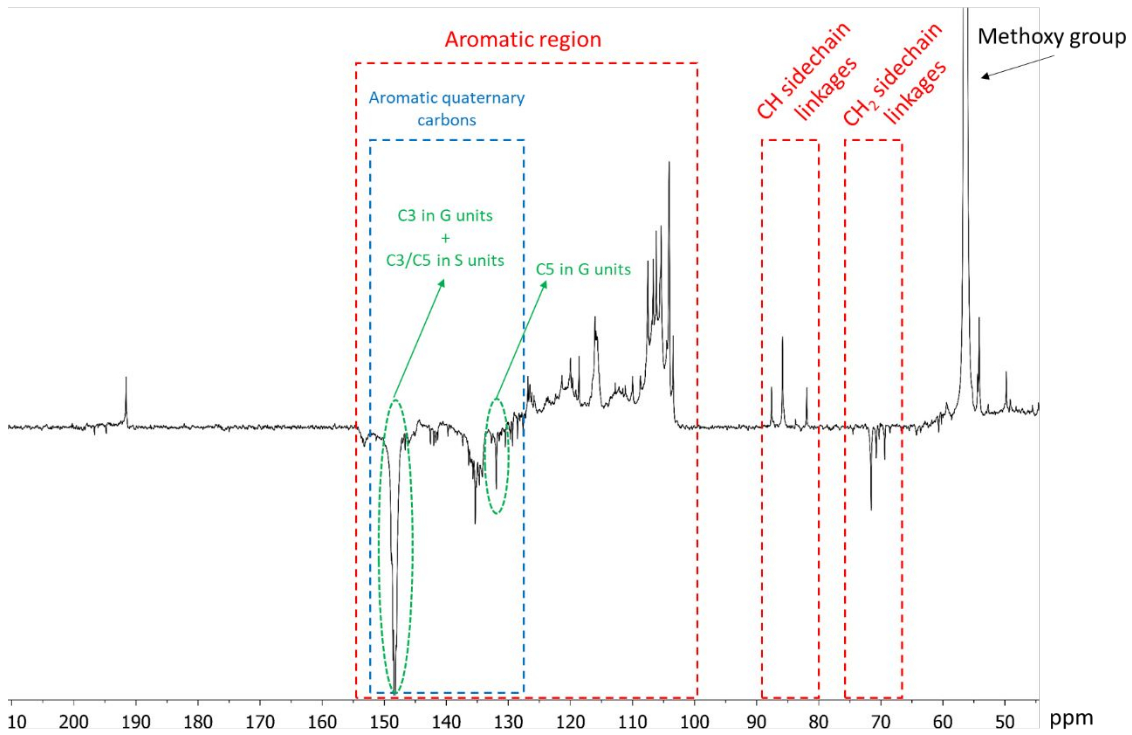

Figure S9: ^{13}C-APT spectrum of the eucalyptus EtOAc fraction (SE_1).

DSC Analysis

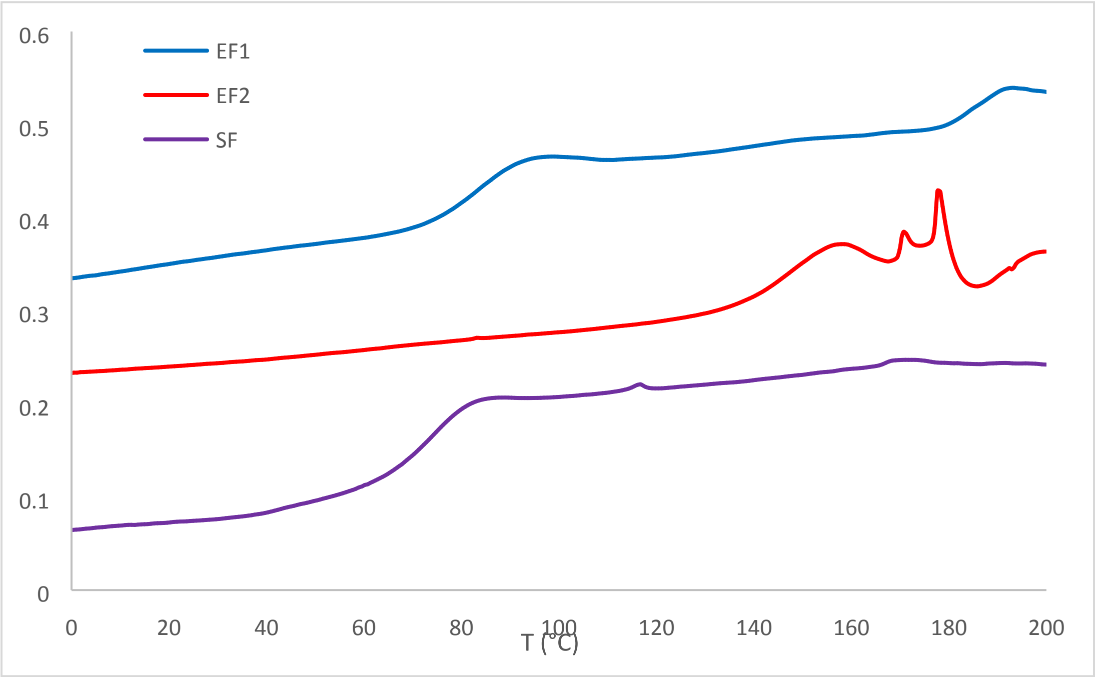

Figure S10: *DSC analysis of the different lignin fractions*

Lignin modification.

HSQC of the epoxidated fractions (DMSO-d$_6$)

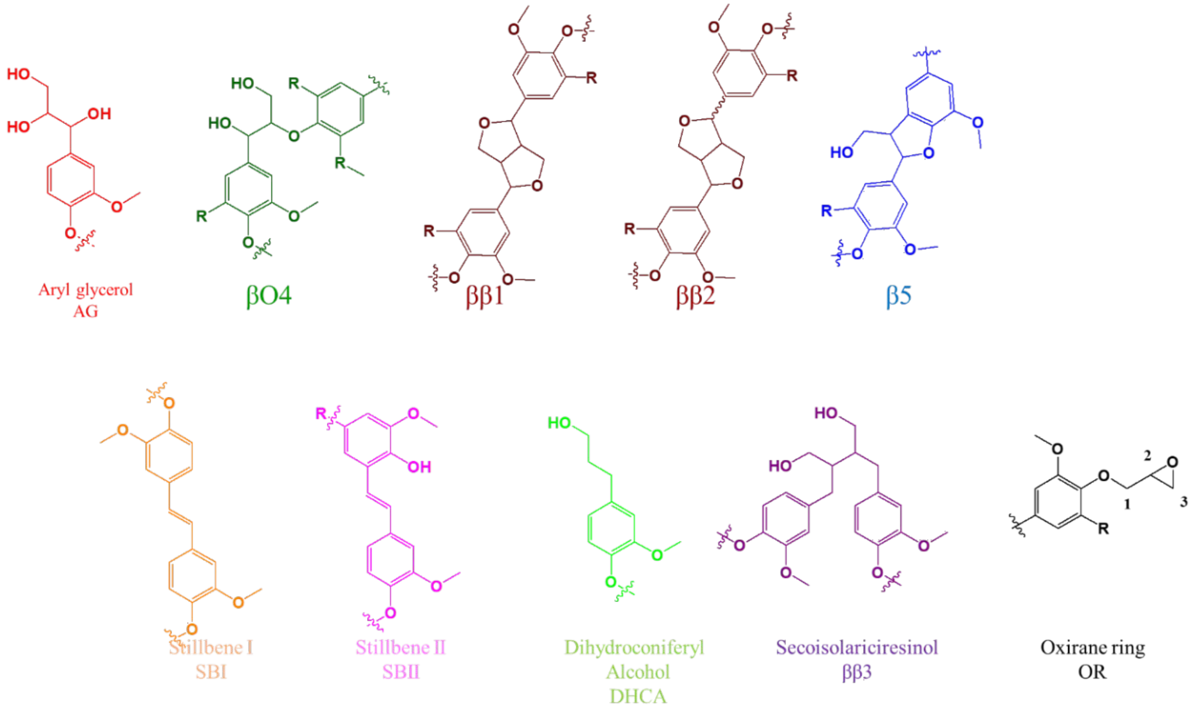

Figure S11: Structures observed in the epoxidated fractions.

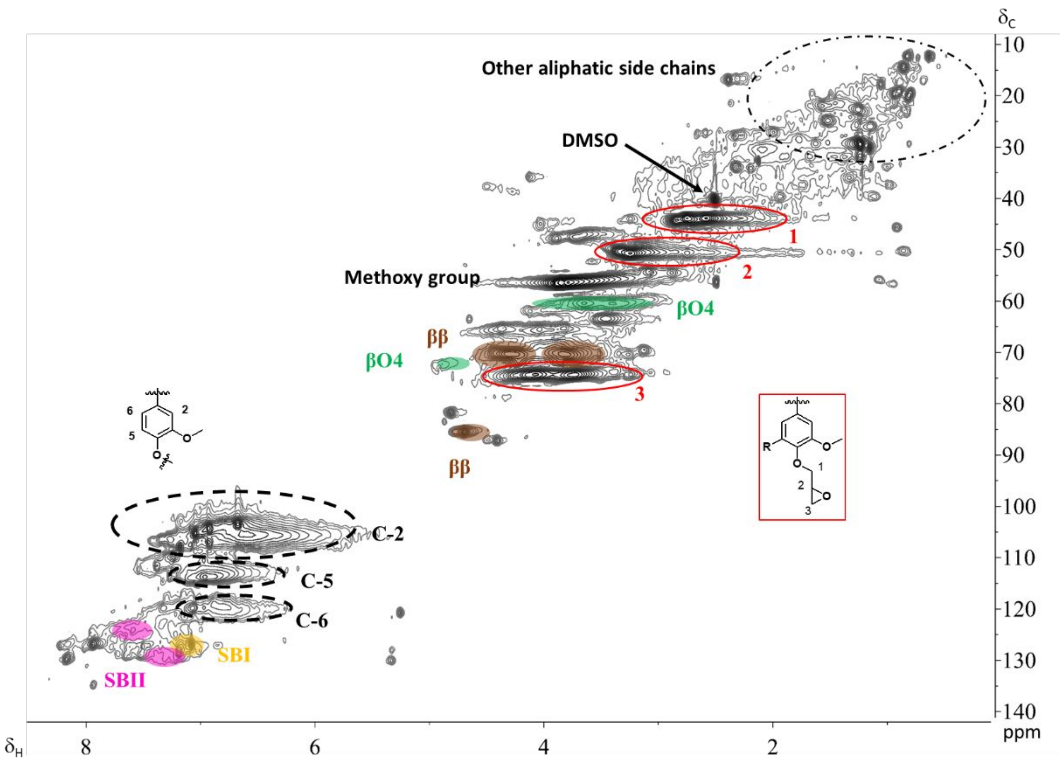

Figure S12: HSQC analysis of the epoxidated eucalyptus EtOAc fraction (EE_1).

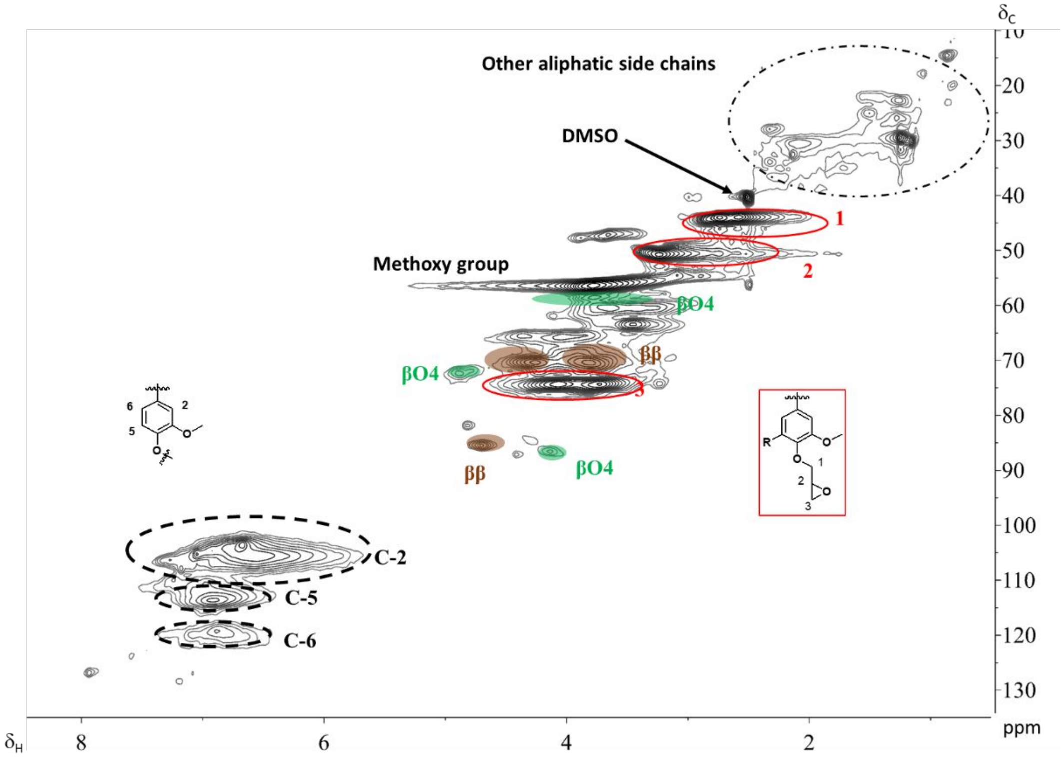

Figure S13: HSQC analysis of the epoxidated eucalyptus EtOH fraction (EE_2).

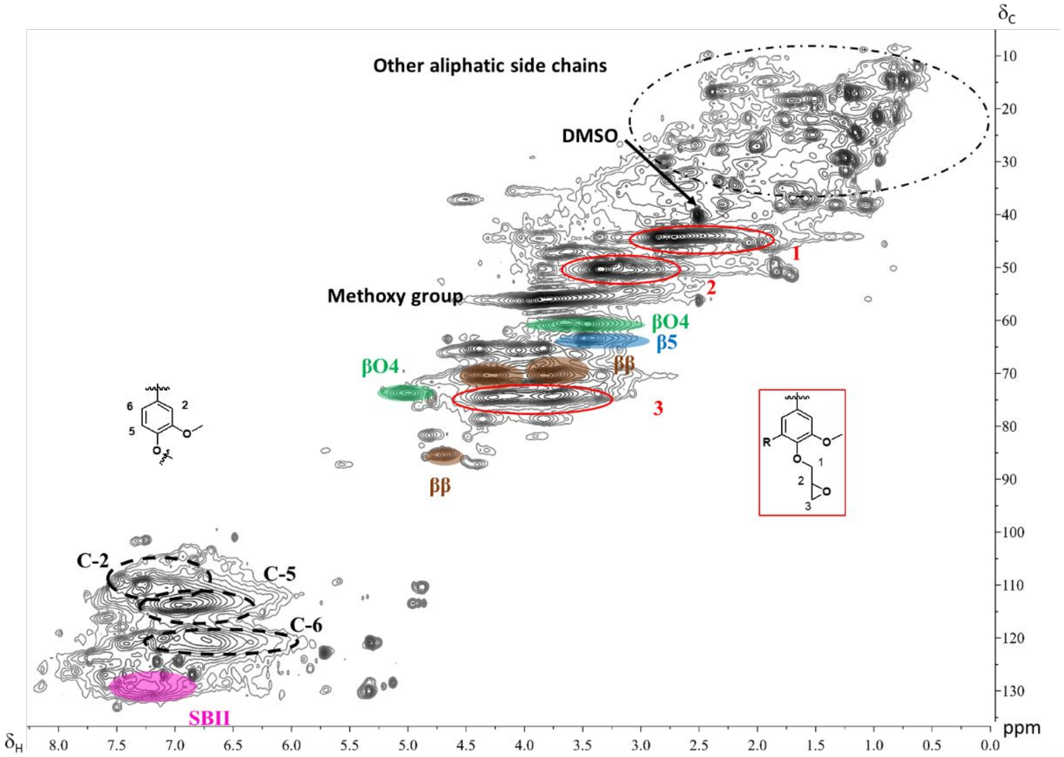

Figure S14: HSQC analysis of the epoxidated spruce EtOAc fraction (SE).

^{31}P-NMR ($CDCl_3$): Typical example of epoxidated lignin fraction

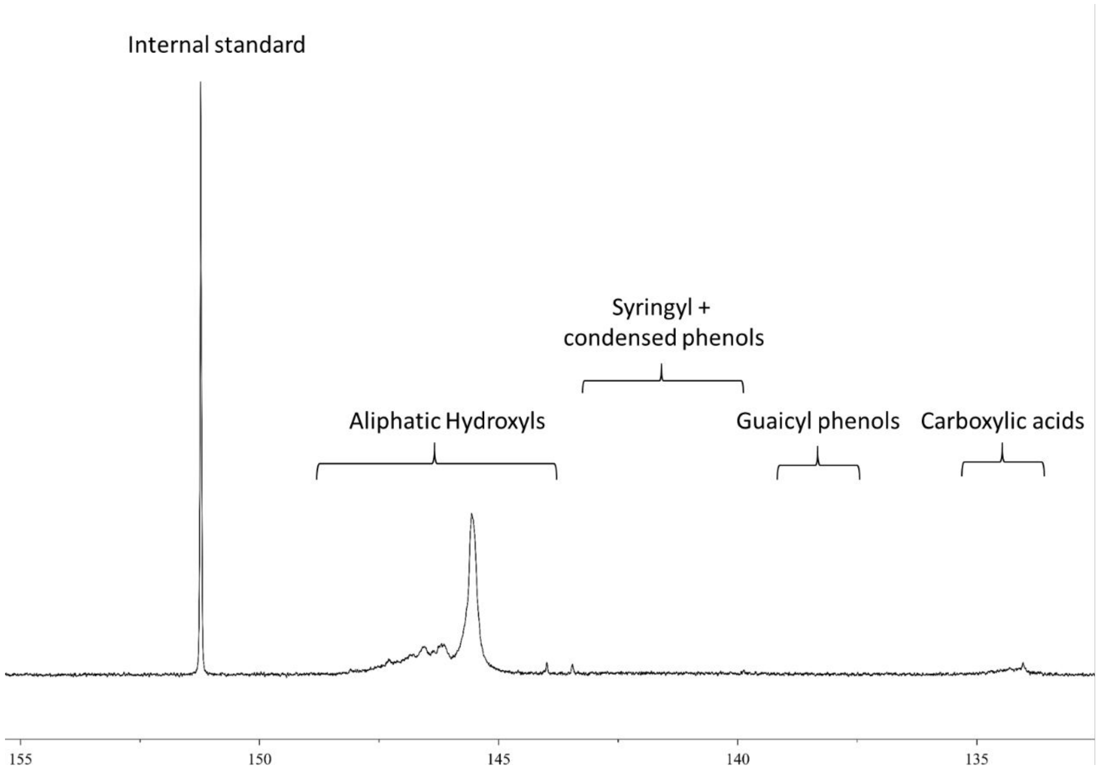

Figure S15: ^{31}P-NMR of the epoxidated eucalyptus EtOAc fraction (EE_1).

^{1}H-NMR quantification of the oxirane rings

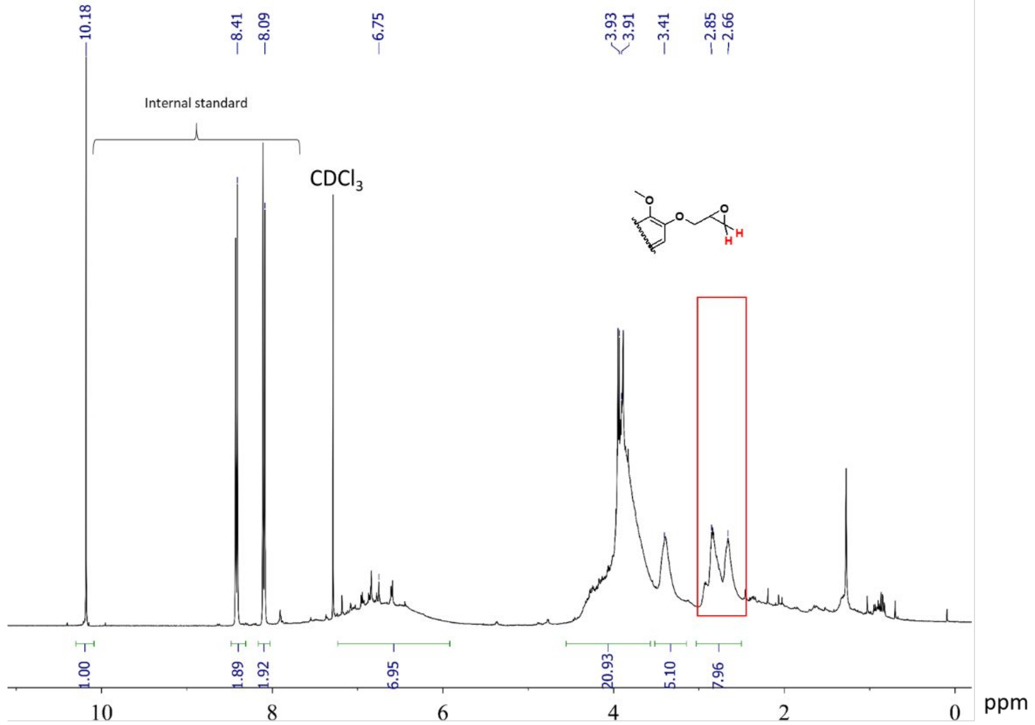

Figure S16: ^{1}H-NMR quantification of epoxy-groups of EE_1 with 4-nitrobenzaldehyde as internal standard.

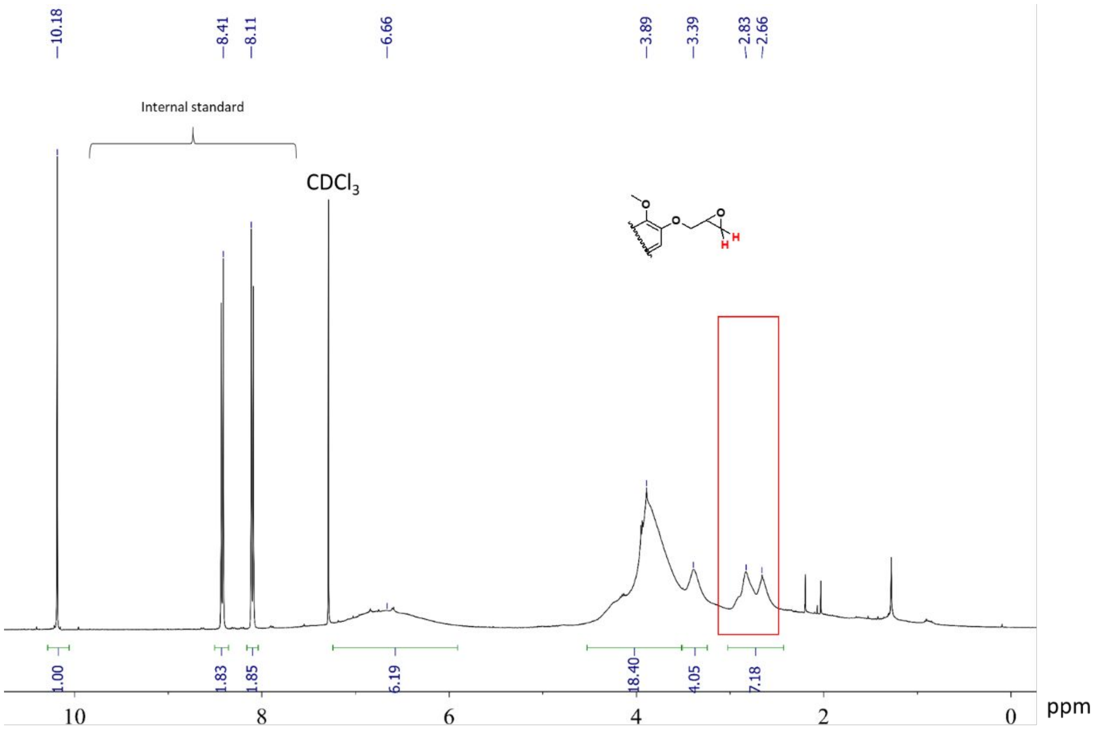

Figure S17: ^{1}H-NMR quantification of epoxy-groups of EE_2 with 4-nitrobenzaldehyde as internal standard.

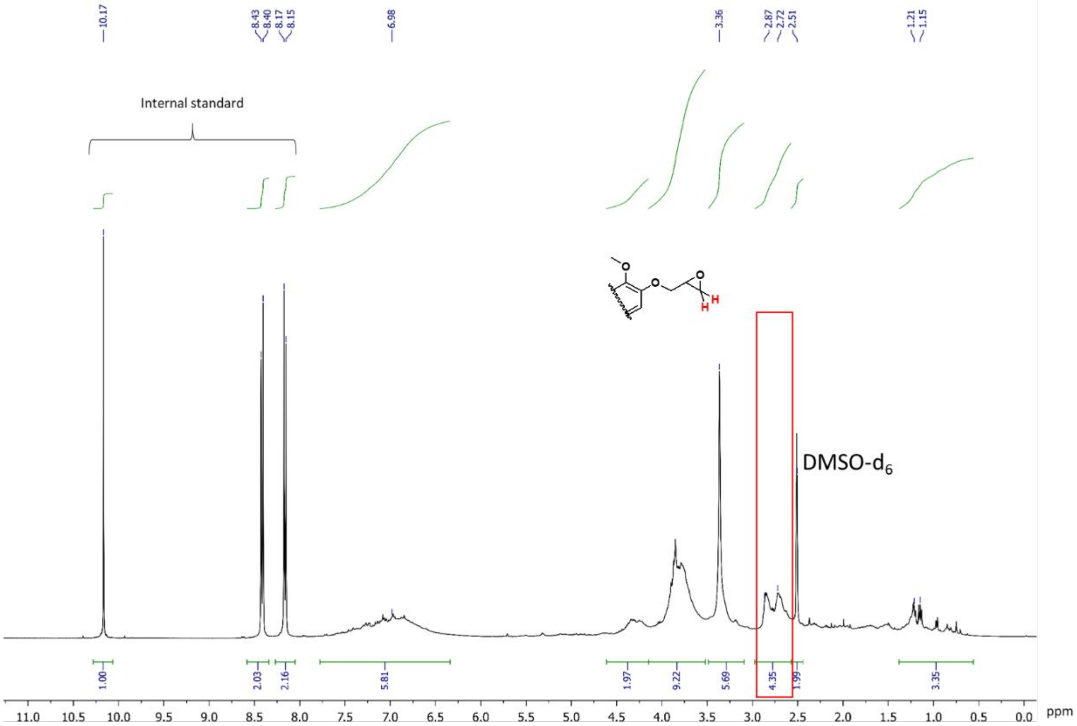

Figure S18: ^{1}H-NMR quantification of epoxy-groups of SE with 4-nitrobenzaldehyde as internal standard.

Characterization of the thermosetting resins.

FTIR Analysis

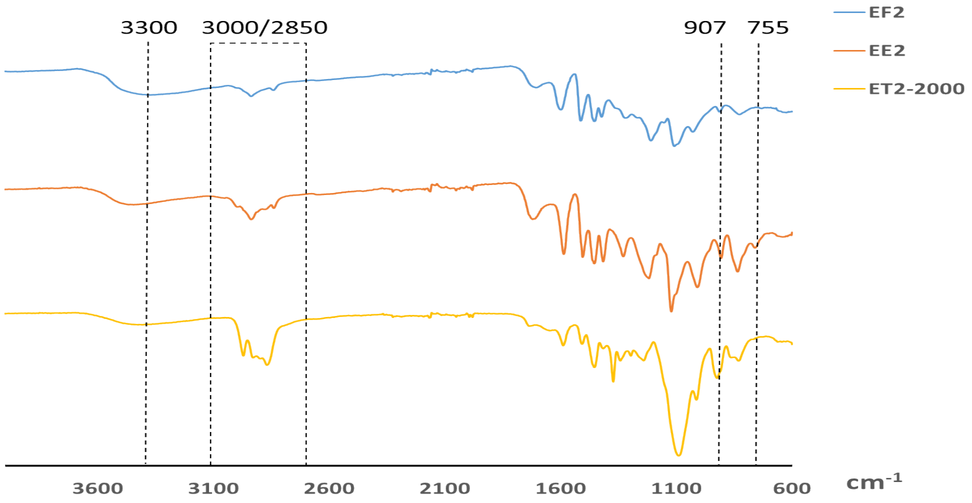

Figure S19: FT-IR analysis comparing the spectra of EF_2, EE_2 and $ET_{2\text{-}2000}$

DSC Analysis

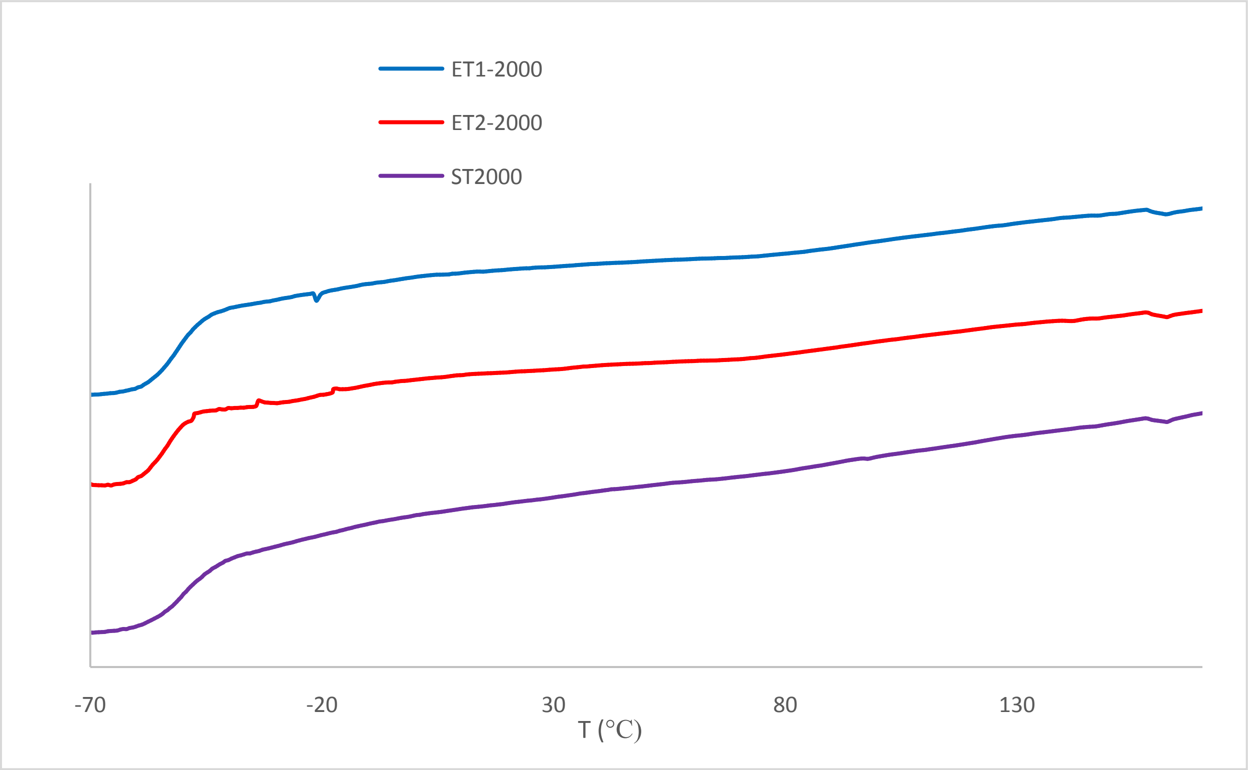

Figure S20: DSC analysis of the resins based on JD 2000.

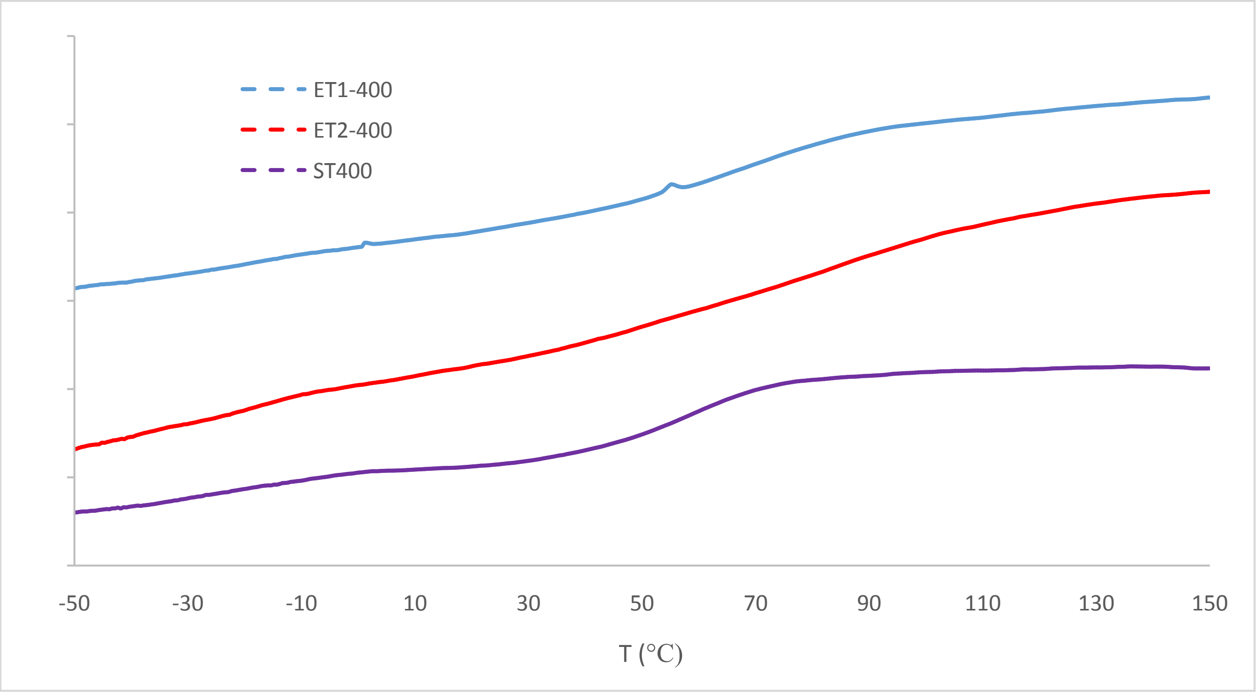

Figure S21: DSC analysis of the resins based on JD 400.

Tensile tests

(The reported curves represent single experiments. The values reported in the manuscript result from the average and standard deviation of multiple tests)

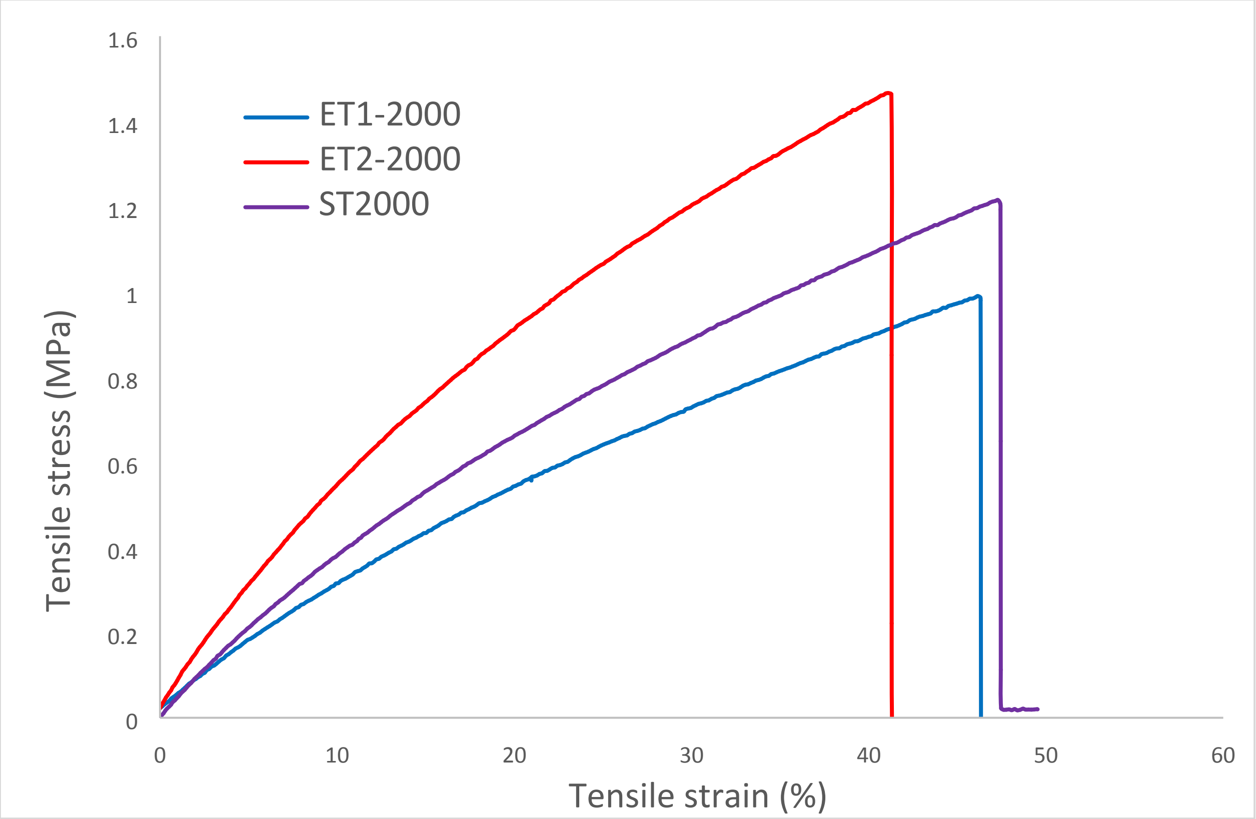

Figure S22: Representative tensile stress-strain curves of the cured resins based on JD 2000

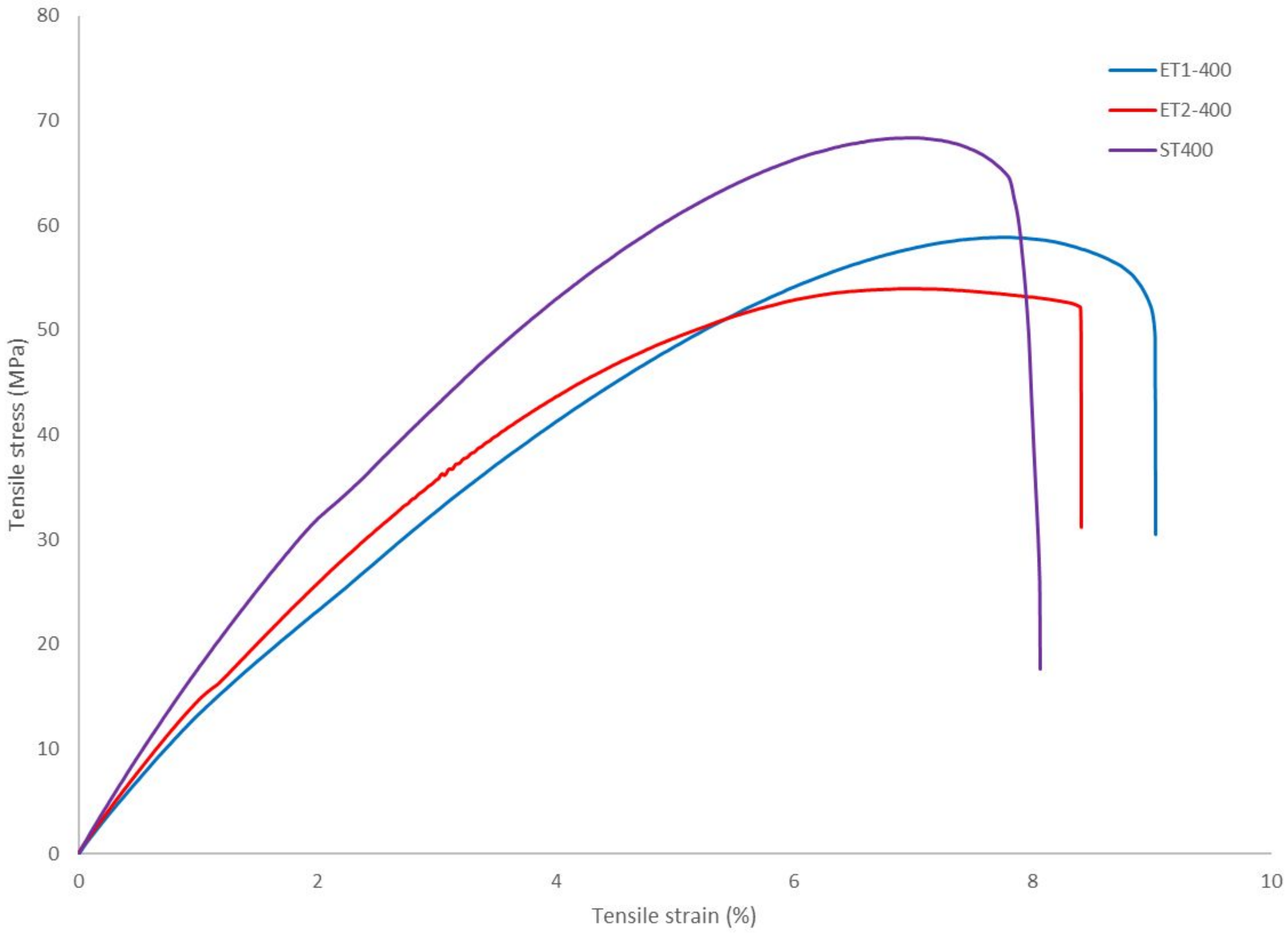

Figure S23: Representative tensile stress-strain curves of the cured resins based on JD 400
